# Early Holocenic and Historic mtDNA African Signatures in the Iberian Peninsula: The Andalusian Region as a Paradigm

**DOI:** 10.1371/journal.pone.0139784

**Published:** 2015-10-28

**Authors:** Candela L. Hernández, Pedro Soares, Jean M. Dugoujon, Andrea Novelletto, Juan N. Rodríguez, Teresa Rito, Marisa Oliveira, Mohammed Melhaoui, Abdellatif Baali, Luisa Pereira, Rosario Calderón

**Affiliations:** 1 Departamento de Zoología y Antropología Física, Facultad de Biología, Universidad Complutense, Madrid, Spain; 2 Instituto de Patologia e Imunologia Molecular da Universidade do Porto (IPATIMUP), Porto, Portugal; 3 CBMA (Centre of Molecular and Environmental Biology), Department of Biology, University of Minho, Braga, Portugal; 4 CNRS UMR 5288 Laboratoire d’Anthropologie Moléculaire et d’Imagerie de Synthèse (AMIS), Université Paul Sabatier Toulouse III, 31073 Toulouse, France; 5 Dipartimento di Biologia, Università Tor Vergata di Rome, Rome, Italy; 6 Servicio de Hematología, Hospital Juan Ramón Jiménez, Huelva, Spain; 7 Faculté des Sciences, Université Mohammed Premier, Oujda, Morocco; 8 Faculté des Sciences Semlalia de Marrakech (FSSM), Université Cadi Ayyad, Marrakech, Morocco; 9 Instituto de Investigação e Inovação em Saúde, Universidade do Porto, Porto, Portugal; 10 Faculdade de Medicina da Universidade do Porto, Porto, Portugal; University of Perugia, ITALY

## Abstract

Determining the timing, identity and direction of migrations in the Mediterranean Basin, the role of “migratory routes” in and among regions of Africa, Europe and Asia, and the effects of sex-specific behaviors of population movements have important implications for our understanding of the present human genetic diversity. A crucial component of the Mediterranean world is its westernmost region. Clear features of transcontinental ancient contacts between North African and Iberian populations surrounding the maritime region of Gibraltar Strait have been identified from archeological data. The attempt to discern origin and dates of migration between close geographically related regions has been a challenge in the field of uniparental-based population genetics. Mitochondrial DNA (mtDNA) studies have been focused on surveying the H1, H3 and V lineages when trying to ascertain north-south migrations, and U6 and L in the opposite direction, assuming that those lineages are good proxies for the ancestry of each side of the Mediterranean. To this end, in the present work we have screened entire mtDNA sequences belonging to U6, M1 and L haplogroups in Andalusians—from Huelva and Granada provinces—and Moroccan Berbers. We present here pioneer data and interpretations on the role of NW Africa and the Iberian Peninsula regarding the time of origin, number of founders and expansion directions of these specific markers. The estimated entrance of the North African U6 lineages into Iberia at 10 ky correlates well with other L African clades, indicating that U6 and some L lineages moved together from Africa to Iberia in the Early Holocene. Still, founder analysis highlights that the high sharing of lineages between North Africa and Iberia results from a complex process continued through time, impairing simplistic interpretations. In particular, our work supports the existence of an ancient, frequently denied, bridge connecting the Maghreb and Andalusia.

## Introduction

Determining the timing, identity and direction of migrations in the Mediterranean Sea throughout human history, the role of “migratory routes” in and among regions of Africa, Europe and Asia and the effects of sex-specific behaviors of population movements all represent current major topics in Paleoanthropology, Prehistory, Archeology, and Population Genetics [1–4].

The geographic proximity between the African and European Mediterranean coasts, mainly at its western end (14 km), has allowed multiple coast-to-coast human movements. As a crucial component of the Mediterranean world, the North of Africa has been dominantly involved in migrations within and beyond the region and has also received migrants from neighboring Mediterranean Europe and southwestern Asia, which contributed considerably to human, cultural and commercial exchanges. Northern Africa is distinguished by its complex prehistory and history, both resulting in a distinctive archaeological record [5]. The prehistoric industries that have been successively registered there (from old Aterian to Capsian, through Iberomaurusian) do not follow a linear replacement sequence but rather create an interesting debate about population continuity or discontinuity [6,7]. The region has also suffered extreme climatic changes, with oscillations between arid phases that did not allow human survival and humid episodes that transformed a part of the bordering Sahara desert into a humid landscape.

Transcontinental migrations are thought to have occurred in several periods across human history, and most researchers set an earlier limit at the end of the Paleolithic [2]. A common argument against prehistoric human flow is based on the dangerous winds, ocean currents and wave patterns created around the Strait that links the Mediterranean Sea directly to the Atlantic Ocean. However, other neighboring eastward areas, such as the *Alboran route*, which is bordered by southeastern Iberia and northeast Morocco, provide for a more secure but longer voyage [1,8]. Clear features of transcontinental ancient contacts have been identified from archeological remains, such as the *Taforalt harpoon*, an Iberomaurusian utensil contemporaneous with the Spanish Magdalenian, detected in northeastern Morocco [9]. Later, strong parallelism in the introduction, adoption and expansion of the *Neolithic package* (the same domesticated species, similar technology transfer, etc.) in southern Iberia and the Maghreb has been interpreted as evidence of tangible strategic relationships between the opposite shores [10,11].

African maternal genes have left imprints both in southern Iberia and the Atlantic façade of the Peninsula [8,12]. Comparatively, markers are less abundant for paternal genes although we have found visible traces of the North African E-M81 Y-chromosome lineage (referred to as the “Berber marker”) in the Andalusian gene pool [13,14]. A recently published study aimed at analyzing the geographic distribution of autosomal immunoglobulin genes at the *GM* locus across the Mediterranean highlighted the relatively high frequency (4% on average) of the sub-Saharan *GM 1*,17 5* haplotype in the Andalusian and neighboring Iberian Atlantic regions compared to other Mediterranean European populations [15]. These findings are understandable given the variation patterns of African mtDNA haplogroups across the Iberian Peninsula. Other genetic studies performed in western Mediterranean people have yielded abundant signals of a notable contribution of maternal Eurasian lineages (for some populations even reaching ~80%) in North Africans [16–19]. These data essentially distinguish the northernmost neighbors from sub-Saharan people [20–23].

The screening of specific mtDNA African lineages in Mediterranean Europe has led to the analysis of whole mitochondrial genomes (mitogenomes), especially in members of the Iberian Peninsula and related Islanders [24–28]. These African mtDNA clades, which have distinctive evolutionary histories, are associated with particular patterns of geographic spread and origins. The distributions of U6 and M1 suggest that they are relevant components of North African populations, with the former being comparatively more represented among northwestern African people and the latter in northeastern Africans (see [29]). The macro-haplogroup L, where the root of the present human mitochondrial phylogeny is located, however, exhibits a particular high incidence and widespread distribution within sub-Saharan Africa, decreasing towards North Africa [30,31].

One of our recently published studies [8] was mainly conducted to ascertain the global variation patterns of mtDNAs in the western and eastern Andalusian region, which are represented by autochthonous people with familiar origins in the Huelva and Granada provinces, respectively. Data showed a high internal genetic diversity accompanied by a significantly heterogeneous African contribution within the Andalusian region: 8.86% and 1.65% of U6 lineages, and 5.70% and 0.83% of L lineages, respectively. These findings and others mentioned above provide scientific support to consider these native populations as prominent targets for studying the transcontinental human migrations and the genetic relationships in the western end of the Mediterranean. The chosen area is complex and is of great strategic importance. Both northern and southern populations surrounding the maritime region of Gibraltar Strait have exhibited markers of reciprocal gene flow.

Accordingly, 62 complete mtDNA sequences from Andalusians and Moroccan Berbers (from Asni, Bouhria and Figuig, see [21]) have been integrated in an updated worldwide comprehensive database of U6, M1 and L lineages to accomplish the following: *i*) to explore the phylogeographical patterns of these major haplogroups and their derived sub-branches, mainly through the Mediterranean region; *ii*) to refine their current phylogenies and estimate coalescent ages; and *iii*) to obtain convincing information on the role of North Africa and the Iberian Peninsula regarding the time of origin, number of founders and expansion directions of these lineages. Inferences on the changes of the past population dynamics based on entire U6 mtDNA sequences have also been proposed. In short, based on the pioneer data provided in this work, we have attempted to improve the understanding of the genetic impact of population movements and demographic events that occurred over the last millennia at the extreme end of the Mediterranean region, mainly underlying the African heritage.

## Results

### mtDNA African lineages in Iberia and surrounding areas. Phylogeographic patterns

Table 1 presents the types and frequencies of the U6, M1 and L mtDNA haplogroups detected in 750 maternally unrelated autochthonous individuals from western and eastern Andalusia. For comparison, mtDNA data from three populations of Moroccan Berbers published earlier by [21] were also analyzed. Western Andalusians from Huelva show a distinctive African influence (11.8% of the total mtDNA variability) compared to those from the eastern part of the region (Granada province) where the proportion of African maternal haplogroups is much less pronounced (3.6%) in determining the population structure (*F*_*ST*_ = 0.0604; *P*-*value* = 0.0090). The significant enlargement of the Andalusian sample set implemented in the present survey in relation to the 279 sequences reported in [8] reinforces the high heterogeneity detected in the variation of global mtDNA between western and eastern Andalusia.

**Table 1:** Types and frequencies of mtDNA African lineages detected among autochthonous Andalusians.

	Western-Andalusia (Huelva, *n* = 280^a^)		Eastern-Andalusia (Granada, *n* = 470)		Asni^b^ (*n* = 53)		Bouhria^b^ (*n* = 70)		Figuig^b^ (*n* = 94)	
	*n*	%	*n*	%	*n*	%	*n*	%	*n*	%
**U6**	**21**	**7.50**	**7**	**1.49**	**6**	**11.32**	**1**	**1.43**	**3**	**3.19**
U6a	18	6.43	5	1.06	5	9.43	1	1.43	3	3.19
U6bd	2	0.71	2	0.43	1	1.89	-	-	-	-
U6c	1	0.36	-	-	-	-	-	-	-	-
**M1**	**1**	**0.36**	**3**	**0.64**	**2**	**3.77**	**3**	**4.29**	**2**	**2.13**
**L**	**11**	**3.93**	**7**	**1.49**	**12**	**22.64**	**9**	**12.86**	**42**	**44.68**
L0	-	-	1	0.21	-	-	-	-	4	4.26
L1b	4	1.43	-	-	4	7.55	5	7.14	6	6.38
L2a	2	0.71	1	0.21	1	1.89	2	2.86	3	3.19
L2b	3	1.07	-	-	2	3.77	-	-	-	-
L2d	-	-	-	-	-	-	-	-	1	1.06
L3b	-	-	-	-	-	-	-	-	8	8.51
L3d	-	-	2	0.43	-	-	-	-	-	-
L3e	-	-	-	-	4	7.55	2	2.86	20	21.28
L3f1b	2	0.71	-	-	-	-	-	-	-	-
L3h1b	-	-	2	0.43	-	-	-	-	-	-
L3x	-	-	1	0.21	-	-	-	-	-	-
L5	-	-	-	-	1	1.89	-	-	-	-
**African lineages (*n***_**t**_ **)**	**33**	**11.79**	**17**	**3.62**	**20**	**37.74**	**13**	**18.57**	**47**	**50.00**
***African lineages (completely sequenced)***	***19***		***13***		***7***		***7***		***16***	

Data from Moroccan Berbers are shown for comparisons.

^a^is the sample size;

^b^from [21].

Within the Iberian Peninsula, the highest frequencies (7.50%) of the U6 haplogroup are registered in Huelva province. Interestingly, Asni Berbers settled in the foothills of the High Atlas Mountains also yielded a much higher proportion of U6 sequences (11.32%) than that observed in related populations from Bouhria (1.43%) and Figuig (3.19%), located in the northeastern flank of Morocco.

The U6a sub-haplogroup is prevalent in southwestern Spain [comparatively, our data reflect the highest values in the Huelva sample (6.43%) and Asni Berbers (9.43%)]. The mean number of pairwise differences (MNPD) within the U6a —based on the mitochondrial HVS-I control region—ranged from 2.0 in southern Iberia ([32,33], present study) to 2.20 in native Moroccan populations [18,21,34–38]. U6bd is much more restricted across mainland Iberia and is observed at very low frequencies (0–1.47%) [32,39,40].

Regarding lineage L, the sub-haplogroups L1 (L1b), L2 (L2a, L2b) and L3 (L3d, L3f, L3h, L3x) again distinguish western (3.92%) from eastern (1.28%) Andalusians. In the analyzed Berber samples, the L1b registered an average of 7% (~6–8%). In Figuig Berbers, L3e appears conspicuously represented (21.3%), which is a finding that is not shared by the other two Moroccan Berber populations (3–8%). The evolutionary origins of the L0, L1, L2 and L3 lineages are located in sub-Saharan Africa [41]. The neat markers of mtDNA L lineages among North Africans again support the view that the Sahara desert has not been an insurmountable barrier to the populations that border it ([15,38,42], among others). The M1 haplogroup is weakly represented in Andalusia <1% (Iberia: 0–2.1%), whereas in Moroccan Berbers, the frequencies vary between 2.1 and 4.3%.

**Fig 1:**
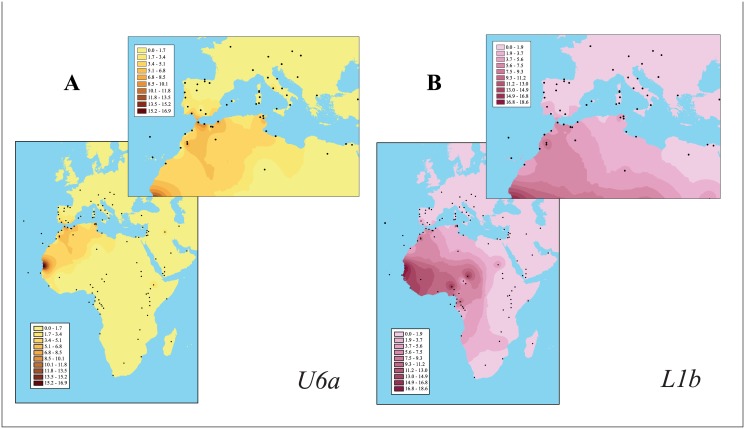
Contour (surface) maps of mtDNA *U6a* (A) and *L1b* (B) sub-haplogroups. Frequency distributions of these maternal lineages in a broad geographic range and the Mediterranean Basin are shown. Frequencies are expressed in percent (see color scale). Population locations and references can be found in Fig A and Table D in S1 File.

Fig 1 presents the spatial distribution patterns of the U6a and L1b mtDNA lineages across the Mediterranean Basin and other selected regions using data sets from a large number of populations (see Fig A in S1 File for sampled localities). As shown in Fig 1A, the highest U6a frequencies are mainly found across Maghreb as well as in Mauritania. The radiation of U6a reaches Europe, mainly through the Iberian Peninsula. The presence of U6a in other northern Mediterranean populations (e.g., Italy) is much lower [43] (for comparison, Fig B in S1 File shows the phylogeography of the U6 haplogroup as a whole). The spatial patterning of L1b (Fig 1B) highlights its strong incidence in West Africa from Guinea Gulf towards northwestern Maghreb. The presence of L1b is perceptible in southwestern Iberia (1.43%, present study; Iberian Peninsula: 0–2.84%) and also in southern Italy and Sicily (0.65–1.09%) [43]. Contour maps of the haplogroup M1 and other L lineages are displayed in Figs C-I in S1 File.

The genetic relationships among mtDNA U6a sequences (observed haplotypes from the HVS-I control region) and separately for the case of L1b were determined, and the median-joining networks are presented in Fig J in S1 File. It is clear a great level of sharing in the major nodes between North Africa and the Iberian Peninsula. The L1b mtDNA network, with a clear star-like branching clade (Fig J-B in S1 File), reveals that the L1b lineage is mostly present in northwestern Africans (with Moroccans the largest contributors) but also in Iberians (e.g., Andalusians from Huelva and Madeira islanders), Italians and Egyptians.

The variation of the U6a and L1b clades and their associated star-shaped networks could be consistent with human population expansions throughout Mediterranean Africa and Europe. However, we have to take into account here the lower molecular resolution level that provide HVS-I sequences compared to that reached by complete sequence information (see next section).

### mtDNA complete sequencing. Phylogeny and coalescence estimates

A more in-depth analysis is necessary to determine the complex history of African maternal lineages, particularly within Iberia and other populations of the northern Mediterranean shore. We have addressed this goal by analyzing the full mitochondrial variation of 32 Andalusian individuals from Huelva and Granada, which are a subset of the entire Andalusian sample (50 of the total 750) bearing the U6, M1 or L haplogroups ([8], present study). Following the same conditions, 30 Moroccan Berber individuals from Asni, Bouhria and Figuig have also been screened. Table A in S1 File shows complete haplotype structures.

**Fig 2:**
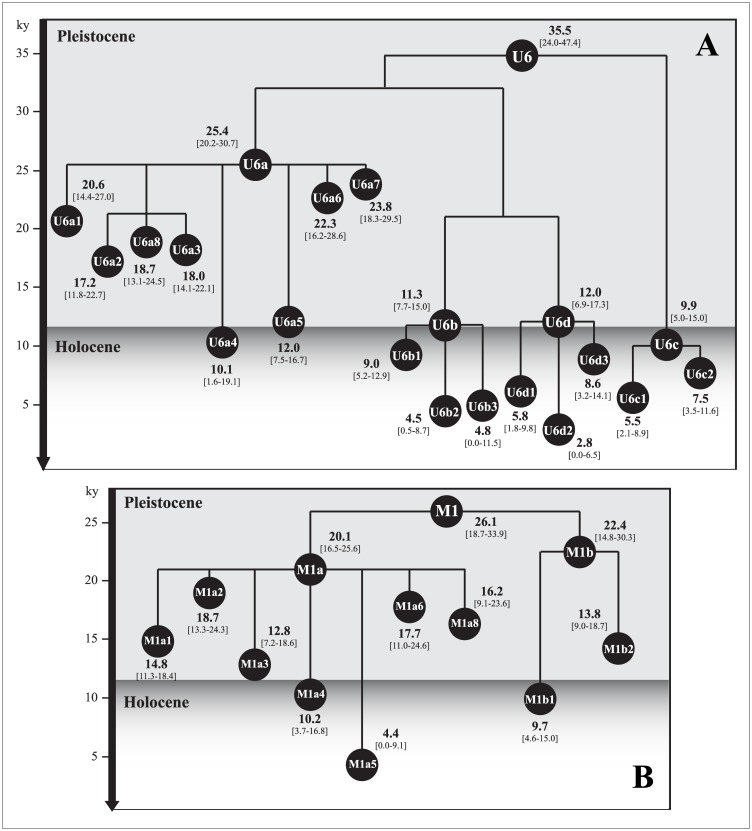
Simplified phylogeny of mtDNA *U6* (A) and *M1* (B) haplogroups. The tree provides maximum likelihood (ML) coalescent estimates (ky, thousands of years) and 95% confidence intervals [95% CI] for major nodes and branches.

Fig 2 presents the schematic inferred phylogenetic trees for U6 and M1 with maximum-likelihood (ML) age estimates (coalescent times) and confidence intervals for all ramifications. In the complete S1–S5 Datasets, refined phylogenetic trees for U6, M1, L1, L2 and L3 based on the search for a high number of mitogenomes published to date (*n* = 2,182; 436 is the mean number of analyzed samples by haplogroup) are provided.

The top node of the mtDNA U6 haplogroup (Fig 2A) coalesces at 35.5 ky (confidence intervals 24.0–47.4), at the Early Upper Paleolithic [44]. Our result is consistent with those previously published by [25,28,29]. The deepest major U6a sub-clade first split at approximately 25.4 ky (20.2–30.7) radiating into several branches and other nested hierarchical clades. U6a is the most frequent U6 clade, with a predominant representation of Maghreb (Moroccan) samples (U6 complete phylogenetic tree in S1 Dataset).

An inspection of the U6 phylogenetic tree (S1 Dataset) showed that it is not easy to infer whether Iberia or North Africa bear more basal lineages. Most sub-haplogroups have lineages descending from the nodes. The most extreme case is the only Moroccan sample, in the root of U6a, whereas the rest are assigned to different sub-haplogroups. U6a6 would be a good North African candidate because most samples described to date have been found in Maghreb (six from Morocco and two from Tunisia). U6a6 dates at 22.3 ky (16.2–28.6), which is a time that matches one of the expansion periods of U6 in North Africa [25]. Within this clade, the two Spanish samples (one from Granada, present study, and one from Asturias) are derived ones, branching from nodes dating back to 4.8 ky (0.7–9.0) and 3.3 ky (0.0–7.3). The most probable introduction occurred from North Africa in a period younger than 4.0 ky.

Looking more effectively at the geographic distribution of samples across the U6 haplogroup, it is possible to affirm only that U6 derived lineages are widespread across North Africa, Iberia, and even Atlantic sub-Saharan Africa, and less so in remaining Europe (see S1 Dataset). Our U6 tree built from mitogenomes shows that U6a1 is predominantly European because it contains a significant number of sequences of Mediterranean individuals mainly from the northwestern shore with a leading Iberian contribution (21 of the 29 European samples) and has an ancestral node in Portugal (accession number HQ651694). Only a few Maghreb sequences form the U6a1 cluster (7/39, 17.95%). U6a2 (17.2 ky [11.8–22.7]) is highly represented in Ethiopia and is scattered in the Near East. U6a3 (18.0 ky [14.0–22.1]) comprises 44 mitogenomes and includes specific sub-Saharan branches, some Iberian and Jews radiations, and a few Near Eastern and Egyptian sequences. Inside the U6a3b cluster and its derivative U6a3b1, mainly Iberian and Maghreb sequences are observed and they show close relationships. The U6c (9.9 ky [5.0–15.0]) and U6d (12.0 ky [6.9–17.3]) are present in Iberia, Europe and North Africa at low frequencies. U6a5 (12.0 ky [7.5–16.6]) is common in sub-Saharan Africa and is present in Tunisia and Italy (two of the six samples are from Sicily) indicating a possible route from Tunisia into Italy even though Tunisian and Italian samples are not in the same sub-branch. Nonetheless, these findings could be related to the destruction of Carthage (149–146 BC) or the Islamic expansion in Sicily (902–1091). U6a7a (7.0 ky [2.4–11.7]) and U6b (11.3 ky [7.7–15.0]) have basal branches everywhere in North Africa, Iberia, Near East and Atlantic sub-Saharan Africa. U6a7 branches have been interestingly linked to historical episodes [28]. The presence of Jewish mtDNA sequences within the U6a7a clade could trace the Jewish (Sephardic) diaspora from Spain (15^th^ century) to other territories in western Europe and outside the continent. One Andalusian from Huelva enriches this cluster with one U6a7a1b sequence (accession number KT819220). Nevertheless, the strongest phylogeographic signal appears to be associated with the U6b1a lineage, yielding a coalescence age of 3.0 ky (1.0–5.1) that is very close to the colonization of the Canary Islands (2.49 ± 0.6 ky) [45]. With a high frequency among native populations, the U6b1a appears to be a sister branch of a Maghreb expansion [28,29].

The M1 simplified tree (Fig 2B) also shows a certain complexity although lower than the U6 tree, and the findings are different in terms of both evolutionary past and geographic distribution. The M1 clade has a younger coalescent time (26.1 ky [18.7–33.9]), followed soon by the radiation of its two descendants, M1a (21.0ky) and M1b (22.4 ky) (see Table B in S1 File). The M1 phylogeny shows more representatives from eastern Africa (Ethiopia and neighbor areas around the Horn of Africa) and outside the Mediterranean Basin (e.g. the Caucasus and southwestern Asia) (see spatial distribution of M1 haplogroup in Fig C in S1 File). Only a few Iberians are scattered across the M1 phylogenetic tree (*n* = 9/114), and they are not clearly associated with the Maghreb sequences. These results suggest some arrivals to the Peninsula linked to minor migrations from northwestern Africa, where M1 registers relevant frequencies (average of 5.34%), but also from the eastern Mediterranean and the Middle East (see M1 complete phylogenetic tree in S2 Dataset).

**Fig 3:**
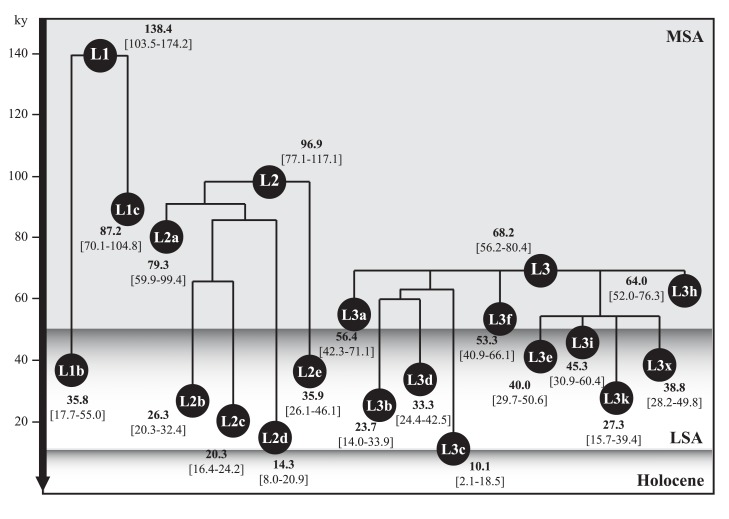
Simplified phylogeny of mtDNA *L* haplogroup. The tree provides maximum likelihood coalescent estimates (ky, thousands of years) and 95% confidence intervals [95% CI] for L1, L2 and L3 sub-haplogroups. MSA: Middle Stone Age; LSA: Late Stone Age.

Coalescent ages for the L-sub-haplogroups (L1, L2 and L3) are shown in the schematic L tree (Fig 3). The main L input in Europe corresponds to L1b, which is the youngest branch (35.8 ky [17.7–55.0]) of its parental clade L1 (138.4 ky [103.5–174.2]) and is contemporaneous with that of the U6 haplogroup. Based on the composition of L1b inside its L1 complete phylogenetic tree (S3 Dataset), western African branches prevail even though some interesting radiations towards northern latitudes are visible.

In the context of the present study, the most remarkable markers of the L lineages within the European gene pool correspond to the sub-clades: L1b1a6 (6.9 ky), L1b1a8 (12.7 ky), L1b1a12 (7.8 ky) and pre-L1b1a16 (10.0 ky). Notably, our L1b1a6 branching structure is different from that presented in [26]. The sub-clade has been further enriched with one Andalusian sequence (from Huelva) and another three from our sampled Moroccan Berbers (one individual from each Berber subpopulation). Other new data provided in the current work have allowed us to focus on L1b1a8 and L1b1a12 phylo2genies, which have also been modified with respect to [26]. The former sub-clade contains mtDNA sequences from only southern and northwestern Spain (Andalusia and Galicia regions, respectively) and Morocco (Figuig Berbers); the latter cluster (L1b1a12) is composed of Iberian and non-Iberian European samples. Furthermore, the pre-L1b1a16 (L1b1a9 for [26]) gathers mitogenomes from the western Mediterranean [Iberian Peninsula (JN214438 and JN214447) and Morocco (EU092667)] and others from central Mediterranean Europe (JN214460, JN225465 and JN225466). Therefore, some L1b clades of the phylogenies would be compatible with an *in situ* evolution within Europe following an input from the African continent as an introduction into Europe/Iberia via North Africa. As most European samples are within L1b1 the initial migration time is limited to the time of this clade (11–13 ky). Other Iberian and non-Iberian European sequences spread over L2 and L3 clades with associated robust phylogeographic signals (see phylogeny trees in S4 and S5 Datasets and contour maps in Fig D-I in S1 File). The position of the Iberian samples within L2 and L3 phylogenies is of considerable complexity. In most cases, Iberian sequences do not cluster with the North Africans sequences but rather with those from the Middle East or Europe. Interestingly, the Spanish samples showed a tendency to group together in the trees although they originate from very distant peninsular regions. It is likely that some of these Spanish mitochondrial sequences may correspond to descendants of emigrants to *Hispanoamerica* who returned to the Peninsula after mixing with Afro-American people. The Portuguese samples are strikingly scarce, and none of them belongs to the L3 mitochondrial haplogroup. The European origin proposed for L2a1k [26,46] with a coalescent age of 10.3–10.6 ky is unclear in our constructed phylogeny because we have ascribed three Berber sequences. Hence, the two parallel branches, the European and Moroccan, obscure the source of this lineage. However, considering the deeper diversity of L2a1 in Africa we can assume a likely movement from North Africa to Iberia.

### African lineages in the Mediterranean: timeframes and dispersals

**Fig 4:**
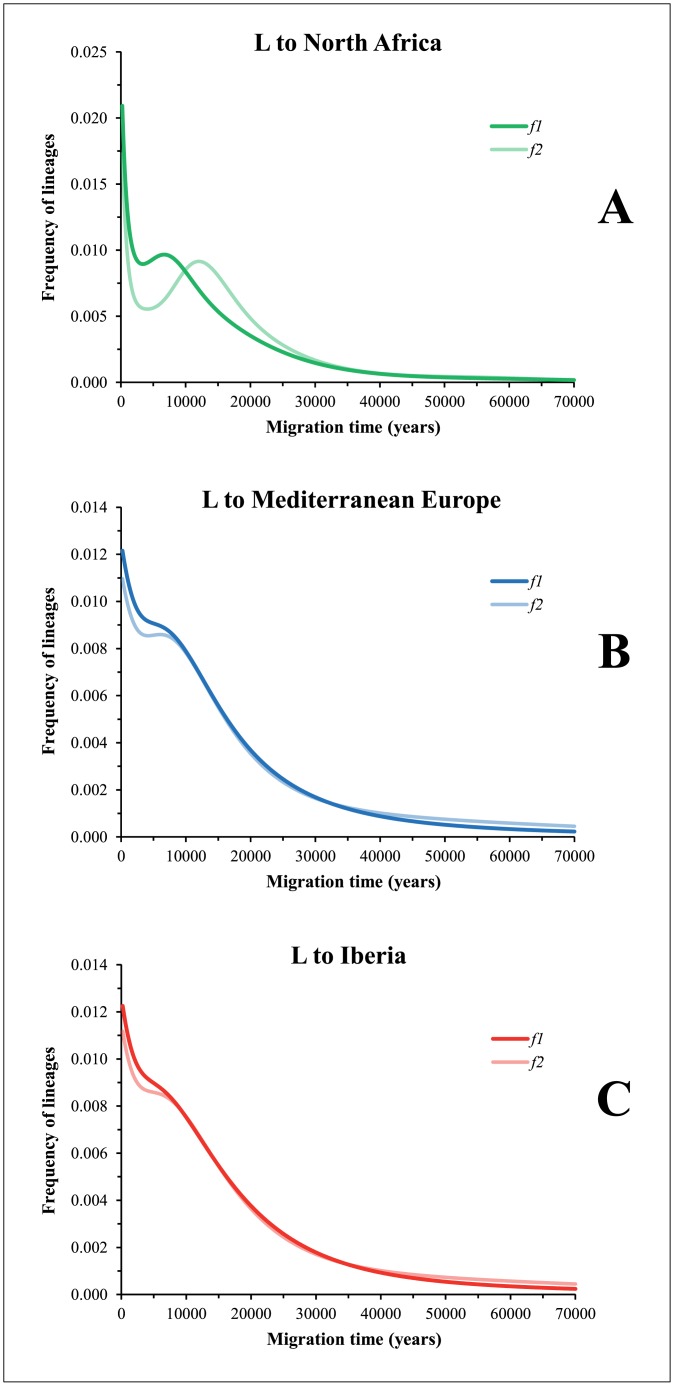
Founder analysis for mtDNA *L* haplogroup. The plots show probabilistic distributions of founder clusters across migration times—using the *f1* and *f2* criteria [39]—for population expansion of mtDNA L lineages into North Africa (A), Mediterranean Europe (B) and the Iberian Peninsula (C). Sub-Saharan Africa is assumed as the source population against North Africa, Mediterranean Europe and Iberia as the sink populations.

The examined phylogenies show old traces of African maternal lineages in the Iberian Peninsula, and help to set upper boundaries for the entrance of these lineages in this region. Nevertheless, in order to establish better time estimates and directionality for migrations across the Mediterranean Basin, we used the founder analysis (FA) methodology. We began by applying FA to the HVS-I diversity, balancing the lower discriminative information of this mtDNA region with the higher number of samples sequenced. Fig 4 displays the plots showing FA results for the L haplogroup. North Africa, Mediterranean Europe and Iberia were assumed to be sink populations against sub-Saharan Africa as the source population. The most intense input of L lineages into North Africa (Fig 4A) seems to have occurred at two time points, one of which was very recent (peak at 0) and the other around the period of the Holocene climatic optimum, HCO (6.6 ky—*f1* criterion—; and 11.8ky—*f2* criterion). When trying to impose two migration periods, the first at 0.5 ky (*f1*) (representing the slave trade from sub-Saharan western Africa to the Maghreb and other contemporaneous events) and the other at 8.0 ky (*f2*) (around the middle of the HCO), it is possible to ascertain (see Fig K in S1 File) that the older period was responsible for the migration of approximately 2/3 of current sub-Saharan L lineages observed presently in North Africa; the slave trade and contemporaneous events there would have introduced 1/3 of this pool. The results for the introduction of L lineages into Iberia or Europe are similar to those, that is, a prehistoric episode would be the main contributor to the sub-Saharan presence in Mediterranean Europe and Iberia (Fig 4B and 4C). A deep inspection of the L-haplogroup founders introduced in North Africa showed that most of them (including basal L1b) reached the area at the older period of migration (Fig L in S1 File). Compared with those L lineages that entered into Mediterranean Europe, the L1b root haplotype clearly dominates the pool of L lineages. More importantly, 79.5% (*f1*) and 81.5% (*f2*) of the lineages that suggest an early Holocene (the Holocene represents the last 11,700 calendar yr before AD 2000 [47]) entrance in Europe also shows the same period of arrival into North Africa, indicating that the migration sequence was likely sub-Saharan Africa—North Africa—Mediterranean North. In fact, we detected genetic closeness between North African and European L-mitogenomes, especially lineages shared with Iberians. In contrast, only 24.2% (*f1*) and 20.3% (*f2*) of the L-lineages that entered Europe in the historic period match the ones that arrived in North Africa, suggesting mainly independent introduction routes. As our data suggest, an entrance of these lineages into Europe directly from sub-Saharan Africa in the historic period is likely related with the slave-trade in Europe.

**Fig 5:**
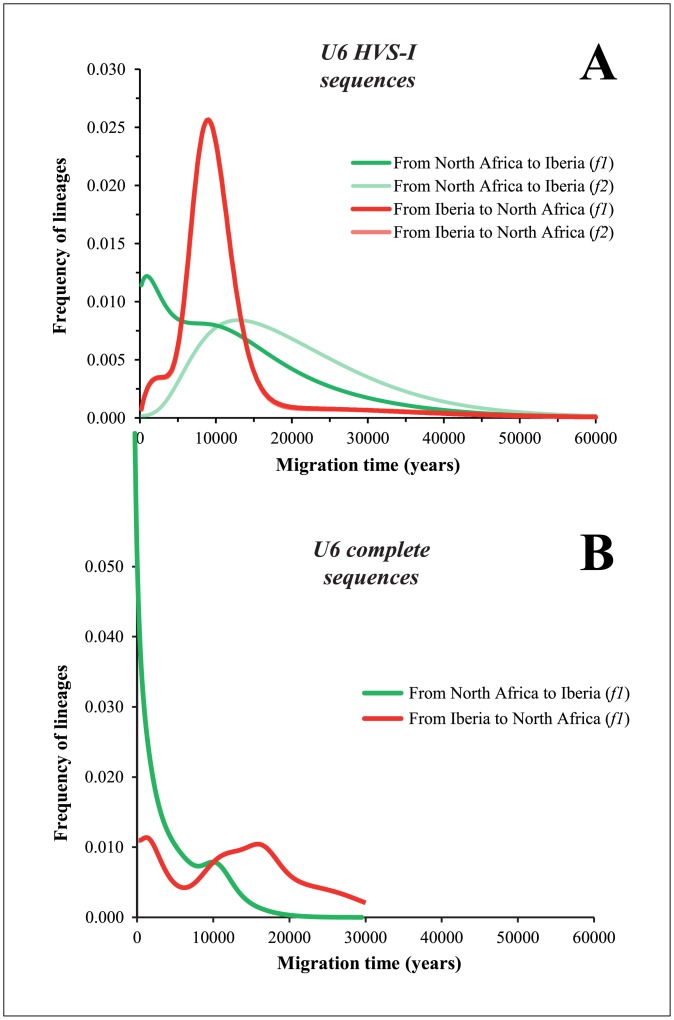
Founder analysis for mtDNA *U6* haplogroup. The plots show probabilistic distributions of U6 founder clusters for HVS-I sequences (A) and complete genomes (B) across migration times scanned at 200-year intervals from 0 to 60 ky.

We began by performing FA for U6 lineages using HVS-I sequences, and the results are displayed in Fig 5A. Assuming the most accepted migration direction from North Africa into Iberia, *f1* criterion shows a main peak at 0.8–1.0 ky BP but preceded by a plateau at approximately 10.0 ky, whereas at *f2*, there is a peak at only 13.0 ky (green line). Migration in the opposite direction, that is, from Iberia to North Africa produces a high peak at 9.0 ky and a slightly lower peak at 3.0 ky, for both the *f1* and *f2* criteria (*f2* curve is covered by *f1* shape). Basically, these results indicate that U6 HVS-I diversity is not particularly informative about which side of the Mediterranean Basin was the most probable source of the migration and which was the sink population for U6. Both haplogroup U6 phylogeny and its geographic distribution gave further support to this statement.

Many U6 polymorphisms located in the HVS-I region (as 16189, 16278, 16311) are highly recurrent, conferring then high-reticulation. Therefore, we attempted to determine whether complete sequences add more information to solve the above issue (Fig 5B). Because North Africa and Mediterranean Europe showed a strong interleaving of lineages within almost all the clades of haplogroup U6, we performed the FA either considering North Africa or Iberia/Mediterranean Europe as the source and the other as the sink in turn. This analysis will allow achieving two purposes: to evaluate which major region displays the higher level of private diversity, which is an indication of the point of origin of the U6 clade and to estimate the time of arrival in the sink population.

When comparing the founders in both sides of the Mediterranean Sea, a deeper level of diversity within North Africa is observed. This information evidences that the most probable direction of migration for them was from North Africa into Iberia. When testing this model (Fig 5B), two peaks of migration are observed: the oldest one at about 10 ky without the suggestion of any private diversity dating further back than 15 ky; and a recent peak, very close to present times. For the oldest peak, the U6a3, U6a6, and U6a8 are the main North African founders.

When testing the inverse direction of migration, from Iberia into North Africa, which is not supported by the ancestrality of clades, a bias introduced in the analysis must be taken into consideration. Generally, not all the diversity in a clade is transported during a migration, which means that the source location will retain higher levels of private diversity in relation to the sink population. Thus, when performing a reciprocal founder analysis, the age of the migration will be older because the retained variation in the first location will be included in the calculations, while in the case for the private variation in the realistic sink population that is limited to the variation generated after the migration. This is precisely the result obtained when testing FA from Iberia into North Africa, as observed in Fig 5B: lineages would have entered North Africa as early as 20 ky, and the probabilistic distribution mostly places the private diversity between 20 and 10 ky with another expected rise in the recent past. So the apparent older date of migration if accepting an Iberia origin reflects, in fact, an earlier expansion of the private clades in North Africa than in Europe. This circular biasing effect on FA was not apparent in previous studies, but we must keep in mind that the high sharing of lineages between North Africa and Iberia results from a complex process continued through time, impairing a simplistic analysis.

The estimated entrance of the North African U6 lineages into Iberia at 10 ky correlates well with other African lineages such as L1b1a6 as shown here and previously [26]. The estimated founder ages of U6 fits well with the founder ages of L lineages, which show a clear ancestry in Africa, and therefore is an indication that U6 lineages moved from Africa to Iberia in the Early Holocene together with L lineages.

**Fig 6:**
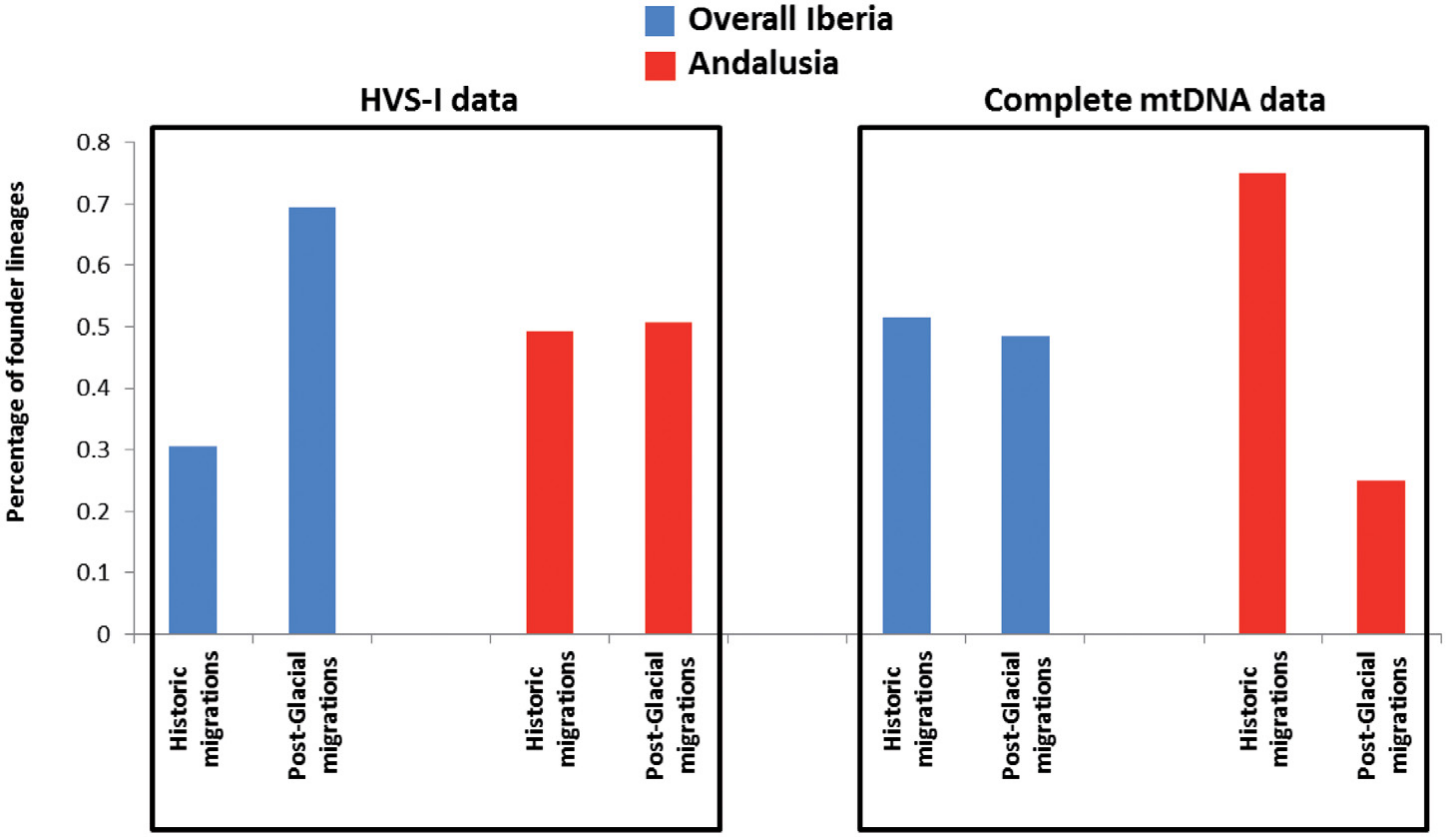
Proportions of mtDNA *U6* founder lineages in Andalusia and overall Iberia. The analysis was based on HVS-I sequences (left side) and entire mtDNA sequences (right side). Two migration periods (historic and post-glacial times) were considered.

We also checked if Andalusia, whose easternmost part, the Granada kingdom was the last Islamic possession in Iberia, would show a different proportion of U6 lineages across the two peaks detected at migration from North Africa, in comparison with overall Iberia (Fig 6). HVS-I based FA attributes an older age to the introduction of the lineages (0.3 to 0.7 of historic to post-glacial events) and similar proportions (0.5) for Andalusia, while the more informative content of complete sequences shows equivalent results (0.5 to 0.5) for the overall Iberian pool,. Mitogenomes show that the Andalusian U6 lineages were mainly introduced in historic times (0.7 fraction).

**Fig 7:**
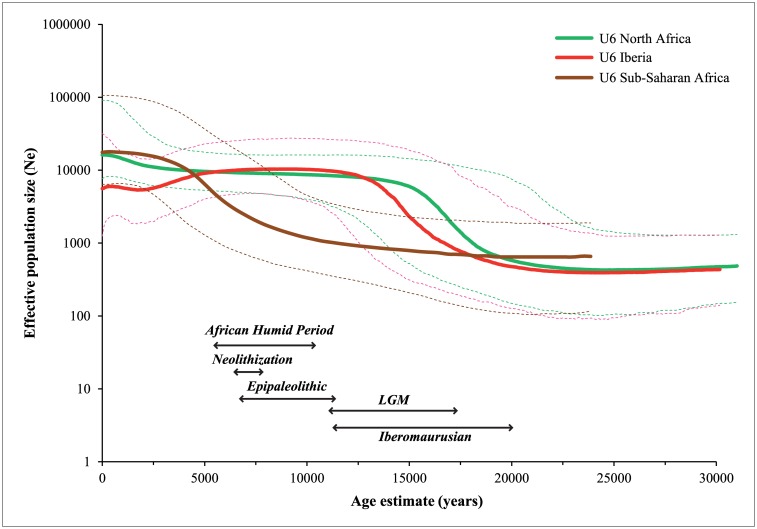
Bayesian Skyline Plot (BSP) analysis of entire mtDNA *U6* sequences. Temporal changes of the effective population size, *Ne* in sub-Saharan Africa (brown color), North Africa (green color), and Iberian Peninsula (red color) are depicted. Solid lines represent the median values for the log10 of *Ne* on the *Y-axis* within each analyzed geographic region. The 95% HPD (highest posterior density) interval is shown for the three distributions (dashed lines).

In an attempt to add more information about which region, North Africa or Iberia shows the oldest signs in terms of U6 diversity, we performed Bayesian Skyline Plot (BSP) analyses for the U6 haplogroup, based on entire mtDNA sequences (see Fig 7). We inferred the temporal trends of effective population size (*Ne*) for the three geographic regions of sub-Saharan Africa, North Africa and Iberia, from the respective datasets of currently observed U6 sequences. As it can be seen, the population expansion of U6 lineages occurs first in the North African dataset, in the post-glacial period, closely followed (around 2 millennia; even so, these dates should be carefully considered, as expansions in sink populations can reflect demographic events in the source, and by the respective confidence intervals) by the expansion of the Iberian dataset. These expansions occurred during the Iberomaurusian (20.0–11.5 ky BP), and were followed by a long-term stability phase. The expansion of the sub-Saharan dataset occurred much latter, within the African Humid Period (10.0–5.5 ky BP), probably following the migration of U6 from North Africa enabled by the climate advantages in this period. Curiously, there is a decline of the U6 lineages in Iberia in recent times (from 5–2.5 ky, and then a plateau is reached), that was not corrected even with an arrival of U6 lineages during the Roman and Islamic rule of the region as indicated by the FA results.

## Discussion

The attempt to discern origin and dates of migration between neighboring regions sharing close genetic affinities has been a challenge in the field of uniparental-based population genetics (see [48]) and is becoming a difficult task also for autosomal genome wide-based studies [49]. The region of the Mediterranean Basin is among the most demanding admixture zones in the globe, due to the possible occurrence of many migrations in both directions, along thousands of years. Notwithstanding, a few pre-historic and historic migration peaks must have taken a predominant role for these migrations, following population expansions and cultural/political dispersions.

Mitochondrial DNA studies have been focused on surveying the H1, H3 and V lineages when trying to ascertain north-south migrations, and U6 and L in the opposite direction, assuming that those lineages are good proxies for the ancestry of each side of the Mediterranean Basin. However, given the low frequencies of U6 lineages across the Basin, and even in most Berber populations, which ancestry they are assumed to represent, the possibility of migration having been north-south across the Mediterranean Sea has not been scientifically addressed. In this work, by increasing considerably the U6 and L complete sequence datasets observed nowadays in Iberia (centered in Andalusia, which displays a significantly high frequency of these lineages) and northwestern Africa, we were able to provide phylogeographic and FA evidence shedding light on this issue.

Usually U6 genetic history is envisioned as a migration from southwest Asia through North Africa [50]. This hypothesis is based on the general origin of haplogroup U sub-clades in Southwest Asia, which is also the center of the geographical distribution of U sub-clades: Europe, India, Central Asia, East Africa and North Africa. Two possible scenarios for the first U6 haplotype (bearing mutations 3348 and 16172) can be advanced: i) these mutations aroused in the founder region but did not leave any genetic legacy in current human populations there; ii) they originated probably somewhere in North Africa, after the arrival of the U6 founder haplotype. Within North Africa U6 is only significantly frequent at its western edge (as well as in South-western Europe). More importantly, all the most basal branches are virtually restricted to that region (U6b, U6c and U6d), what could indicate its western origin. Nevertheless, it cannot be excluded the major sub-clade U6a, which shows a richness of sub-clades in Northwest Africa [29] although a few of derivative branches also include sequences from East African and the Middle Eastern populations (e.g. U6a2).

A possible explanation for the almost restriction of U6 in the west of North Africa is its virtual disappearance from Egypt, given the continuous gene flow from the Near East as well as the wide physical barrier of the Libyan desert between the Nile Delta and Tunisia. Thus, it renders Egyptians genetically closer to Eurasians than to other North Africans, as shown for the nuclear genome [51]. Nevertheless, as we have confirmed here in enlarged trees based on complete sequence information, that U6b, U6c and U6d branches are intertwined between Iberia and North Africa, most basic phylogeographic inference tools are inefficient to ascertain their origin. Thus, from a purely phylogeographic approach, a third possible scenario would be theoretically possible that U6 emerged in Europe from the root of haplogroup U, as seems to have occurred for U5 [52], but, in opposition to this sister haplogroup, U6 occupied a very restricted area in Europe. Additionally, the U6 distribution would need to be very restrictive even within the Iberian Peninsula, since this area was a probable source of migrations to the remaining Europe during the post-glacial period and Neolithic [53,54], and U6 is basically absent outside Mediterranean Europe. These considerations, *per se*, imply that although not impossible, an origin of U6 in Europe was unlikely. The major increment of U6 at around 20 ky detected in our BSP analysis could better fit the emergence of the Iberomaurusian industry in the Maghreb [7,25] than any known occurrence in Europe/Iberia, also suggesting an evolution of U6 in North Africa. The U6 FA performed here based on complete sequences corroborates that North Africa has a higher level of private genetic diversity.

Still, our FA results of U6 haplogroup have revealed evidence of U6 back-migration from Iberia to North Africa. A southward migration from Iberia is also signaled by some sub-branches of mtDNA lineage H [16,19]. Several and solid evidences, including archaeological and historical data, support a scenario of presumable bidirectional gene flow between North Africa and Iberia. Close parallelisms held between the Upper Paleolithic industries Iberomaurusian and the Spanish Magdalenian (~20–12.9 ky cal BP) [55] would have been the result of exploration and side-to-side human movements. Both the Strait of Gibraltar and the Alboran sea (a probable alternative maritime route, eastern Strait) would have represented key scenarios for explaining those early human contacts [1,11]. Moreover, the Neolithization of southern Iberia and the Mediterranean Maghreb (7.6–6.9 ky) has been considered as the same integrative process [10,11]. The historic episodes (the shared government rule of Iberia and the Maghreb during the Roman Empire and the westward expansion of Islam) strengthen the links between both shores of the western Mediterranean. In ancient times, human movements through the regions were dependent on favorable phases in environmental conditions, which were neither numerous nor lasting.

The pattern inferred for L (especially L1b) lineages provides additional support to ancient migrations towards Iberia in the post-glacial period. L lineages are undoubtedly of sub-Saharan origin, and FA shows their entrance into North Africa between 10–15 ky. Within Africa, geographic distances among its densely populated centers are considerable, and areas, which can be suitable for human settlement, are scarce. Many of the routes connecting major dense regions are separated by mostly depopulated (unoccupied) areas (e.g. between Ethiopia and Nigeria), and some of them pass through the great physical barrier of the Sahara desert (e.g. between the Niger Basin and Morocco and the Nile Delta and Tunisia) that could be crossed only under suitable environmental conditions. Early and long-scale human migrations through the Sahara/Sahel region to North Africa have been associated with the MIS 3 (Marine Isotope Stage 3, ≈50–45 ky BP) and the AHP. The most western river system that ran north across the Sahara to the Mediterranean, the *Irharhar*, was the probable route for human migration connecting western and northwestern Africa [56]. Other probable early migration routes from eastern to northwestern Africa extended across western Africa, not bordering the Mediterranean fringe. These L lineages arrived in Iberia soon after (the oldest peak is at 10 ky), and FA seems to support the role of North Africa as an intermediate post, as most of the lineages (~80%) arrived in Iberia in this period match the ones arrived in North Africa a little time before.

Thus, the most parsimonious model for the oldest demographic events and migrations across the Mediterranean Basin, based on mtDNA evidence, is the following: after 20 ky, U6 lineages had an extensive population expansion in northwest Africa, associated with the emergence of the Iberomaurusian industry in the Maghreb; this pool was further enriched by sub-Saharan L lineages, especially L1b, which began to arrive in North Africa in the beginning of the African Humid Period (AHP, ~11–5.5 ky BP) [57]; U6 and L lineages were introduced from northwest Africa into Iberia in the post-glacial period, most probably by the time of the Younger Dryas/beginning of the Holocene. The opening of the trans-Saharan communications with the African Humid Period, also allowed the southern migration of U6 sequences, and its local expansion as detected in the BSP for lineages observed in sub-Saharan Africa.

The recent migration peaks identified in the FA of U6 and L lineages could be associated with the Islamic rule of Iberia and the slave trade period, respectively. The HVS-I FA attributes a comparatively lower proportion of these recently introduced sequences into Iberia when compared with the post-glacial one: 1/3 vs 2/3, respectively. The analysis performed by [26] on the phylogeography of L sequences observed in Iberia and remaining Europe pointed to 65% of its introduction in recent times (Romanization period, Islamic expansion and Atlantic slave trade), and 35% at older times, as earlier as 11ky. The complete sequence based FA for U6 agrees more with these results, attributing a half-half proportion of sequences in both periods, showing the higher resolution of complete mitogenomes. The historical-based expectation of a higher proportion of newly introduced U6 lineages in Andalusia was confirmed: 70% against 30% in the earlier migration.

Date estimates for migrations across the Mediterranean Basin and the Sahara desert based on mtDNA data do not generally match the ones inferred by genome-wide information, these last tending to be considerably younger. A recent study [58] estimated that the migration from sub-Saharan Africa into North Africa occurred 40 generations ago (≈1.2 ky), matching trans-Saharan slave trade, while their estimate for the back-to-Africa settlement of North Africa (comparison with Near Eastern/European pools) was before 12 ky. On the other hand, [59] showed that the 1–3% African ancestry observed in southern Europeans had an average mixture date of 55 generations ago, matching North African flow at the end of the Roman Empire and subsequent Arab migrations. And even improved methods, allowing to disentangle between several migration events, such as the one developed in [49], conduct to dates of sub-Saharan African admixture in the range 890 to 1754 CE, again pointing to the Islamic expansion and slave trade. The current limitation on date estimates for admixture based on genome wide diversity, due to the phenomenon of recombination, shows the continued usefulness of uniparental markers to shed light on the human evolution history, spanning 200,000 years. In particular, our work supports the existence of an ancient, frequently denied, bridge connecting the Maghreb and Andalusia, located just across the Strait of Gibraltar but also possibly in a more eastern position with a pier on the small island of Alboran.

Here, we have deeply inspected the history of female African genes in the Mediterranean based on demographic, archaeological and paleoclimatic evidences. Further genomic approaches will help us to deepen our understanding of peopling processes in this geographic area. In this context, a suitable sampling process and sample selection, efforts in data compilation for comparative purposes and powerful statistical tools are needed to accurately assess the timing of migratory episodes between North Africa and Europe and their actual impact on the European gene pool, the role of physical barriers as well as the most plausible dispersal routes. Certainly, the region encompassing the western end of the Mediterranean will continue to be of strategic importance for addressing these points.

## Materials and Methods

### Populations and sample selection

We analyzed the mitochondrial genome of carefully selected samples from southern Spain (Andalusia, *n* = 32) and North Africa (Moroccan Berbers, *n* = 30) belonging to lineages U6, M1, L1, L2 and L3. The Spanish subjects are components of our available Andalusian sample stock, which consists of a high number of autochthonous and unrelated individuals (*n* = 750) with family origins—up to the third generation—from the west (Huelva province) and east (Granada province) side of Andalusia. The same strategy for assessing autochthony was applied to the Moroccan Berbers (*n* = 217) [21]. The collection of blood samples and the design of the sampling process was directly conducted by the authors RC (Andalusians) and JMD (Moroccan Berbers). Information about localities and the sampling procedure can be found in [8] and [21]. The Bioethics Committee of the Complutense University of Madrid (Spain) and that at the Université Paul Sabatier III of Toulouse (France) approved human sampling collection for Andalusia and Morocco, respectively, as well as molecular protocols to be used. Written informed consent was obtained from all participants.

An enlargement of the sample set further explored the African genetic influence into Andalusia with respect to that previously studied by [8]. For this purpose, we designed two TaqMan SNP Genotyping Assays (Life Technologies, Foster City, CA, USA) for the detection of U6 (3348 [A/G]) and L/M samples (10873 [T/C]) (see Table C in S1 File). Thus, 122 new subjects from Huelva province and 349 from Granada were tested for African-associated lineages. Combining these samples with those from our previous survey (*n =* 279), 750 Andalusian samples have been screened for the presence of African lineages, among which we found 50 individuals belonging to haplogroups U6, M1 and L. From these 50 samples, we selected a subset of 32 Andalusian subjects for complete mtDNA sequencing –19 from Huelva and 13 from Granada–that presented distinctive control region haplotypes. The large total sample size (*n* = 750) together with the procedure of complete sequence selection would assure a substantial representation of African lineage diversity in southern Iberia. Likewise, mtDNA genomes of 30 Moroccan Berbers belonging to three populations (seven Asni, seven Bouhria and 16 Figuig) were also sequenced. Again, the selection of these 30 samples from a total of 80 subjects was based on control region information.

### Mitogenome sequencing and haplogroup assignment

The mitochondrial genomes were amplified using 32 overlapping fragments [60]. Sequence reactions were run in a 3100 DNA Analyzer (Life Technologies, Foster City, CA, USA) at the Sequencing Service of IPATIMUP (Porto, Portugal). Checking procedures—including resequencing of fragments due to ambiguous base calls—followed previously described indications [25]. Polymorphisms were scored with respect to reference sequence rCRS [61]. BioEdit v.7.2.2 [62] was used for the sequence alignment and editing. Samples were ascribed to haplogroups and sub-haplogroups using the most updated phylogenetic data available at the PhyloTree website [63]. Positions 16182C, 16183C and 16519, *indels* and *heteroplasmy* were omitted for the subsequent phylogenetic analyses. The 62 new complete sequences were deposited in the GenBank database (accession numbers KT819205–KT819266).

### Statistical analyses

Contour (*surface*) maps were constructed to obtain a broader overview of the geographic distribution patterns of the main African mtDNA clades, using ArcGIS Desktop 10.1 software (Redlands, CA: Environmental Systems Research Institute) (more details in [8] and [25]). For this purpose, a number of 3,661 HVS-I control region sequences (see data and references in Table D in S1 File) were collected from a total database of 34,229 samples from Iberia, Europe, Near/Middle East and Africa. The HVS-I sequence compilation (range 16,051–16,400 of rCRS, [61]) comprised 317 U6 sequences, 361 from M1 and 2,983 from certain L sub-haplogroups (L1b, L2a, L2b, L3b, L3d, L3f, L3h1b). All populations considered in this database have a sample size ≥50. For the assignment of geographic coordinates, we considered specific population information recorded in the literature when indicated; otherwise, capital cities were considered. When more than a population sample is available from a specific region, the geographic center was identified using ArcGIS 10.1 “Mean Center” tool. Map templates were taken from http://www.naturalearthdata.com/. It is worth noting that African population samples selected for contour maps (Fig A in S1 File) are dispersed following the present spatial distribution patterns in the continent [64].

Phylogenetic relationships among U6a (*n* = 209) and L1b (*n* = 175) HVS-I sequences were obtained by performing specific median-joining (MJ) networks [65]. A set of populations originating in strategic regions such as mainland Iberia, Italy and major islands, and Africa were used. In addition, diversity values (mean number of pairwise differences intra-lineages) of U6a and L1b sub-haplogroups were calculated.

For phylogenetic reconstruction based on complete mtDNA sequences, we compiled 2,182 mitogenomes (see S6 Dataset). The U6 phylogeny was based on the 20 new sequences reported here and other 246 previously published. M1 analyses were based on our five new sequences and other 109 samples taken from the literature. Finally, L phylogeny comprised 1,802 complete sequences, including our 37 new samples. Macro-haplogroup L was split into L1, L2 and L3 sub-clusters. Network approaches were employed for an initial tree reconstruction [65], and the phylogeny was then refined most parsimoniously by hand. Ambiguities in tree building were resolved by weighting the mtDNA mutations with respect to their occurrences in the global human phylogeny [66]. Because *indel* phenomena were not considered, our reconstructed phylogeny did not match with PhyloTree [63] in some punctual cases (e.g., the association among the sub-branches L3b, L3c, L3d and L3f). mtDNAGeneSyn software was used to convert files [67].

The time to the most recent common ancestor (TMRCA) for haplogroups and sub-haplogroups was estimated using the ρ statistic (for the whole genome and for only synonymous variants) and by maximum likelihood (ML). Complete sequence compilation for age estimates is detailed in S6 Dataset. For ρ coalescence age calculations, we used an estimated mutation rate for the mitochondrial genome of 1 substitution/3,624 years, and a synonymous mutation rate of 1 substitution/7,884 years [66]. Purifying selection was taken into account using the calculator provided in this paper. Standard errors were estimated as in [68]. Maximum likelihood estimates were obtained using PAML 3.13 software [69], considering the HKY85 mutation model with gamma-distributed rates (approximated by a discrete distribution with 32 categories). The ML distances calculated were then transformed into time by means of the same previously mentioned clock.

### Founder Analysis and Bayesian Skyline Plots

To analyze the timing and directionality of human migrations between sub-Saharan Africa, North Africa and Europe, we employed founder analysis (FA) devised by [39]. The method assumes a strict division between hypothetical source and sink populations and two criteria (*f1* and *f2*) for identifying founder sequences to partly account for homoplasy and back-migrations between regions, and thus ensuring that sequence matches are not at the tips of the source phylogeny. The first step was to reconstruct the HVS-I network in the range of 16,051–16,400 bp of the reference sequence (see data used in Table D in S1 File). Then, we identified founders and descendants using an in-house computer tool [70]; and finally, we estimated the age of the migration of each founder using the ρ statistic, assuming an HVS-I mutation rate of one mutation every 16,677 years [66]. Uncertainty estimates were calculated as described previously [31,71]. To display the overall patterns of migration, we calculated the probabilistic distribution of lineages across 200-year intervals.

To test gene flow, FA was performed using information from the U6 and L lineages considering several scenarios. Firstly, we analyzed the arrival of L lineages from sub-Saharan Africa into North Africa, Mediterranean Europe (Iberia, Italy and Greece), and more specifically, Iberia. It is advisable to use all of Africa as the source population for L sequences as most of the founders are in sub-Saharan Africa. Reduced median networks were built using the HVS-I dataset mentioned above. Hypothetical founder were identified in the networks by searching for shared nodes or haplotypes between the hypothetical source and sink populations. The two criteria, *f1* and *f2*, postulates that shared nodes or haplotypes are only considered founders if the source population displays at least one (*f1*) or two (*f2*) derived branches from the hypothetical founder in the source population. Private diversity in each founder will be converted into time using rho and the molecular clock mentioned above. Each founder will be probabilistically allocated to the different hypothetical migration times using a Bayesian approach defined previously [39]. In this regard we took two approaches; one was to defined 200-year intervals between migrations from 0 to 50 ky in order to see the overall distribution of founder ages without any predefined model. This provides a scan that could allow to better define models of migration [31,71]. Using this information as well as paleoclimatological, archaeological and historical data we defined models of migration using predefined migration times. This allows to statistically estimate the percentage of lineages that are associated with each of the stipulated migrations. Throughout the analyses performed two time depths seemed more associated with migrations, one on the early Holocene (defined as 8 ky) and more recent migrations (defined as 0.5 ky). A simplistic model using these two migration times will allow to allocate founder lineages to one of the migration times and define the percentage of lineages that entered in each of them.

Both sides of the Mediterranean Basin were evaluated independently as source areas for U6 sequences in order to ascertain the more probable source and sink population as done before for haplogroups R0a, HV1 and R2 regarding an Arabian or Near-Eastern origin for these [72]. Because the M1 haplogroup has a minor contribution (regarding frequencies and geographic distribution) as African influence in Europe, it was omitted from these analyses.

We also accomplished FA with U6 complete sequences considering North Africa, and Iberian/Mediterranean Europe as the source and the other as the sink and vice-versa, using an *f1* criterion. Because the current FA requires a single value of mutation rate, a time-dependent mutation rate curve is not implemented; we used a central value of the mutation rate between the younger and older estimated founders as performed previously [48]. The mutation rate used was one mutation every 2,643 years for the complete mtDNA genome. In terms of European founder age, we calculated values for the Iberian Peninsula only and for all of Mediterranean Europe.

Finally, to evaluate changes in the effective population size (*Ne*) through time in three key geographic areas (sub-Saharan Africa, North Africa and Iberia), we obtained Bayesian Skyline Plots (BSPs) for U6 complete sequences. See codes and references for the mitogenomes used in S6 Dataset. We used a relaxed molecular clock (lognormal in distribution across branches and uncorrelated between them), the HKY model of nucleotide substitutions with gamma-distributed rates, assuming a generation time of 25 years to rescale the vertical axis of the BSP and using BEAST 1.4.6 [73]. We ran 50,000,000 iterations with samples drawn every 1,000 MCMC steps, after a discarded burn-in of 5,000,000 steps (more details in [25,31]).

## Supporting Information

S1 Dataset U6 Tree. U6 phylogeny built using 266 mitogenomes. Accession number details (sample information and references) are provided in S6 Dataset. The studied Andalusian and Berber mtDNA complete sequences are highlighted in boldface.
(XLS)

S2 Dataset M1 Tree. M1 phylogeny built using 114 mitogenomes. Accession number details (sample information and references) are provided in S6 Dataset. The studied Andalusian and Berber mtDNA complete sequences are highlighted in boldface.
(XLS)

S3 Dataset L1 Tree. L1 phylogeny built using 422 mitogenomes. Accession number details (sample information and references) are provided in S6 Dataset. The studied Andalusian and Berber mtDNA complete sequences are highlighted in boldface.
(XLS)

S4 Dataset L2 Tree. L2 phylogeny built using 706 mitogenomes. Accession number details (sample information and references) are provided in S6 Dataset. The studied Andalusian and Berber mtDNA complete sequences are highlighted in boldface.
(XLS)

S5 Dataset L3 Tree. L3 phylogeny built using 674 mitogenomes. Accession number details (sample information and references) are provided in S6 Dataset. The studied Andalusian and Berber mtDNA complete sequences are highlighted in boldface.
(XLS)

S6 Dataset Database of 2,182 complete mitochondrial DNA genomes. (XLS)

S1 File Supporting figures and supporting tables related to the work. (PDF)
