# Supplementary material for: Early Holocenic and Historic mtDNA African Signatures in the Iberian Peninsula: The Andalusian Region as a Paradigm
Source: PLoS One. 2015 Oct 28;10(10):e0139784. doi: 10.1371/journal.pone.0139784 (PMC4624789; doi:10.1371/journal.pone.0139784)
Supplement: S1 File — (PDF) [file pone.0139784.s007.pdf]

Supporting Information

Early Holocenic and historic mtDNA African signatures in the Iberian Peninsula: the Andalusian region as a paradigm

Candela L. Hernández, Pedro Soares, Jean M. Dugoujon, Andrea Novelletto, Juan N. Rodríguez, Teresa Rito, Marisa Oliveira, Mohammed Melhaoui, Abdellatif Baali, Luisa Pereira & Rosario Calderón

Supporting Tables **2**

Table A. Complete mtDNA haplotypes of Andalusian and Moroccan samples 2

Table B. TMRCA estimates of U6, M1 and L lineages 8

Table C. TaqMan Genotyping Assays design 9

Table D. Database of HVS-I sequences 10

Supporting Figures **14**

Figure A. Geographic location of populations used for contour maps 14

Figure B. Spatial distribution of haplogroup U6 15

Figure C. Spatial distribution of haplogroup M1 16

Figure D. Spatial distribution of haplogroup L2a 17

Figure E. Spatial distribution of haplogroup L2b 18

Figure F. Spatial distribution of haplogroup L3b 19

Figure G. Spatial distribution of haplogroup L3d 20

Figure H. Spatial distribution of haplogroup L3f 21

Figure I. Spatial distribution of haplogroup L3h1b 22

Figure J. U6a and L1b specific median-joining networks 23

Figure K. Probabilistic proportion of L founder clusters in two migration events 24

Figure L. Graphical depiction of the frequency of L founders in two migration events 25

Other Supporting files

S1 Dataset (.xls). U6 Tree. U6 phylogeny built using 266 mitogenomes

S2 Dataset (.xls). M1 Tree. M1 phylogeny built using 114 mitogenomes

S3 Dataset (.xls). L1 Tree. L1 phylogeny built using 422 mitogenomes

S4 Dataset (.xls). L2 Tree. L2 phylogeny built using 706 mitogenomes

S5 Dataset (.xls). L3 Tree. L3 phylogeny built using 674 mitogenomes

S6 Dataset (.xls). Database of 2,182 complete mitochondrial DNA genomes

Table A. Complete mtDNA haplotypes of Andalusian and Moroccan Berber samples. Haplogroups are indicated as reported in respective trees (S1-S5 Datasets). Base change is shown only in transversions and indels. GenBank accession number is provided.

Code	Accession number	Population	Haplogroup	Haplotype
H090	KT819205	Western Andalusia (Huelva)	L1b1a12a	73 152 182 185T 195 198 247 263 315insC 357 522delCA 709 710 750 769 825A 1018 1462 1738 2352 2706 2758 2768 2885 3308 3594 3666 3693 4104 4769 5036 5046 5393 5655 6548 6827 6989 7028 7055 7146 7256 7389 7521 7867 8248 8468 8655 8701 8860 9540 10398 10688 10810 10873 11002 11719 12519 12705 13105 13506 13650 13789 13880A 14178 14203 14560 14766 14769 15115 15326 16126 16187 16189 16223 16264 16270 16278 16293 16311 16400 16519
H088	KT819206	Western Andalusia (Huelva)	L1b1a6	73 151 152 182 185T 195 247 263 315insC 357 523delAC 709 710 750 769 825A 1018 1738 2352 2706 2758 2768 2885 3308 3594 3666 3693 4104 4769 5036 5046 5393 5655 6548 6827 6989 7028 7055 7146 7256 7389 7521 7805 7867 8248 8468 8655 8701 8860 9540 9755 10398 10688 10810 10873 11719 12519 12705 13105 13506 13650 13789 13880A 14110 14178 14203 14560 14766 14769 15115 15326 16126 16187 16189 16223 16264 16270 16278 16293 16311 16519
H092	KT819207	Western Andalusia (Huelva)	L2a1	73 143 146 152 195 263 309insC 315insC 750 769 1018 1438 2416 2706 2789 3594 4104 4769 5460 7028 7175 7256 7274 7521 7771 8206 8701 8860 9221 9540 10115 10398 10873 11719 11914 11944 12693 12705 13590 13623 13650 13803 14566 14766 15218 15301 15326 15784 16093 16189 16192 16223 16260 16278 16294 16309 16390 16519
H093	KT819208	Western Andalusia (Huelva)	L2a1c6	73 143 146 152 189 195 263 309insC 315insC 523delAC 750 769 1018 1438 2416 2706 2789 3010 3594 4104 4164 4769 6663 7028 7175 7256 7274 7521 7771 8206 8701 8860 9201 9221 9540 10115 10398 10873 10954 11719 11914 11944 12693 12705 13590 13650 13803 14566 14766 15301 15326 15784 16169 16223 16239 16278 16294 16309 16390
H095	KT819209	Western Andalusia (Huelva)	L2b1a	73 150 152 182 195 198 204 263 315insC 418 523delAC 750 769 1018 1438 1442 1706 2332 2358 2416 2706 3594 4104 4158 4370 4767 4769 5027 5231 5331A 5814 6026 6131 6713 7028 7119 7256 7521 7624A 8080 8206 8387 8419 8701 8860 9221 9540 10115 10398 10828 10873 11719 11944 12236 12705 12948 13590 13650 13924 14059 14569 14766 15110 15217 15301 15326 16114A 16129 16213 16223 16278 16294 16355 16362 16390
H094	KT819210	Western Andalusia (Huelva)	L2b3a	73 146 150 152 182 195 198 204 207 263 315insC 750 769 1018 1438 1442 1706 2332 2358 2416 2706 3594 4104 4158 4185 4370 4767 4769 5027 5331A 5744 5814 6713 7028 7256 7521 7624A 8080 8206 8387 8701 8860 8925 9221 9540 10115 10398 10873 11719 11944 12236 12705 12948 13590 13650 14059 14544 14766 15110 15217 15236 15301 15928 15944insT 16093 16114A 16129 16213 16223 16271 16278 16390
H159	KT819211	Western Andalusia (Huelva)	L3f1b1	73 189 200 263 315insC 750 1438 1822 2706 3396 4218 4769 5601 7028 7819A 8527 8701 8860 8932 9540 9950 10398 10873 11440 11719 12705 14766 14769 15301 15326 15514 15944delT 16209 16223 16292 16295 16311 16519
H161	KT819212	Western Andalusia (Huelva)	M1a1b1	73 195 263 309insCC 315insC 489 750 813 930 1438 2706 3705 4769 6446 6671 6680 7028 7853 8701 8860 9540 10398 10400 10873 11719 11809 12346 12403 12705 12950C 14110 14766 14769 14783 15043 15301 15326 16129 16183C 16189 16223 16249 16311 16359 16519

H140	KT819213	Western Andalusia (Huelva)	U6a1a1a	73 263 309insC 315insC 523delAC 750 1438 2706 3348 3969 4172A 4769 7028 7805 8860 11467 11719 11938 12308 12372 14179 14766 14927 15326 16172 16183C 16189 16219 16239 16278
H162	KT819214	Western Andalusia (Huelva)	U6a1a1a	73 263 309insC 315insC 750 1438 2706 3348 3969 4172A 4769 7028 7805 8860 9185G 11467 11719 11938 12308 12372 14179 14766 14927 15326 16093 16172 16183C 16189 16219 16239 16278 16291
H139	KT819215	Western Andalusia (Huelva)	U6a1a1a	73 263 309insC 315insC 750 1438 2706 3348 3969 4172A 4769 7028 7805 8860 9185G 11467 11719 11938 12308 12372 14179 14766 14927 15326 16172 16183C 16189 16219 16239 16278 16291
H143	KT819216	Western Andalusia (Huelva)	U6a3b	73 146 152 185 188 263 309insC 315insC 750 1211 1438 2706 3348 4769 7028 7805 8860 11268 11467 11719 12308 12372 13431 14142A 14179 14766 15326 15634 15790 16172 16183C 16189 16219 16278
H144	KT819217	Western Andalusia (Huelva)	U6a3b	73 146 152 185 188 263 309insC 315insC 750 1211 1438 2706 3348 4769 7028 7805 8860 11268 11467 11719 12308 12372 13431 14142A 14179 14766 15326 15634 15790 16172 16183C 16189 16219 16278
H165	KT819218	Western Andalusia (Huelva)	U6a3b1	73 146 152 185 188 263 309insC 315insC 750 1211 1438 2706 3348 4769 7028 7805 8860 11268 11467 11719 12308 12372 13431 14179 14766 15326 15634 15790 16172 16183C 16189 16219 16278 16311
H167	KT819219	Western Andalusia (Huelva)	U6a3b1	73 146 152 185 188 263 309insC 315insC 750 1211 1438 2706 3348 4769 7028 7805 8860 11268 11467 11719 12308 12372 13431 14179 14766 15326 15634 15790 16172 16183C 16189 16219 16278 16311
H137	KT819220	Western Andalusia (Huelva)	U6a7a1b	73 150 152 263 315insC 750 794A 1193 1438 1692T 2706 3348 4769 5120 5471 7028 7805 8473 8860 11467 11719 12308 12372 14179 14766 15043 15326 15530 15632 16172 16219 16278
H134	KT819221	Western Andalusia (Huelva)	U6c2b	73 150 194 263 315insC 437 522delCA 750 793 1438 2706 3348 3688C 4769 4965 5081 6340 7028 8860 11013 11467 11719 12076 12308 12372 13879 14308 14766 15244 15326 16169 16172 16189
H147	KT819222	Western Andalusia (Huelva)	U6d3a	73 263 315insC 750 1438 2706 3348 4336 4769 7028 8860 10397 11467 11719 11947 12308 12372 12501 12530 14518 14766 15326 16172 16174 16188 16219 16311
H168	KT819223	Western Andalusia (Huelva)	U6d3a	73 263 315insC 750 1438 2706 3348 4336 4769 7028 8860 10397 11467 11719 11947 12308 12372 12501 12530 14518 14766 15326 16172 16174 16188 16219 16311
G123	KT819224	Eastern Andalusia (Granada)	L2a1b	73 143 146 152 195 263 309insCC 315insC 750 769 1018 1438 2416 2706 2789 3594 4104 4769 7028 7175 7256 7274 7521 7771 8206 8701 8838 8860 9221 9540 10115 10143 10398 10873 11650 11719 11914 11944 12406 12693 12705 13590 13650 13803 14566 14766 15301 15326 15784 16093 16129 16189 16223 16278 16294 16309 16390
G124	KT819225	Eastern Andalusia (Granada)	L3d1b1	73 150 152 263 309insC 315insC 522delCA 750 921 1438 2706 3459 4769 5046 5147 5605 6272 6680 6842 7028 7424 8618 8701 8860 9156 9540 10398 10873 11719 12705 13105 13886 14284 14766 15301 15326 16093 16124 16223 16311
G125	KT819226	Eastern Andalusia (Granada)	L3d3b	73 152 263 315insC 523delAC 750 921 1393 1438 1719 2706 4688 4769 5147 5773 6722 7028 7389 7424 8277 8279 8618 8701 8860 9540 10398 10873 11719 11779 12046T 12705 13105 13752 13886 14284 14766 15061 15301 15326 16124 16223
G126	KT819227	Eastern Andalusia (Granada)	L3h1b1a	73 152 189C 194 195 263 309insC 315insC 522delCA 750 1438 1719 2706 3777 4388 4769 5300 6359 7028 7717 7861 8152 8701 8860 9509 9540 9575 10044 10398 10873 11590 11719 12705 14410 14766 15301 15326 16179 16223 16243 16256A 16284 16311 16320 16519

G127	KT819228	Eastern Andalusia (Granada)	L3x2b1	73 150 194 200 249delA 263 315insC 494 650 750 1438 2706 3483 4769 5656 5899insC 6401 7028 7933 8158 8311 8584 8701 8817 8860 9254 9540 9941 10398 10819 10873 11719 12705 13708 14766 15301 15326 15519 15758 15928 16169 16193 16195 16223 16243 16261
G086	KT819229	Eastern Andalusia (Granada)	M1a2a1	73 152 195 263 315insCCC 489 513 750 813 1438 2706 4769 6446 6671 6680 7028 8701 8860 9540 10398 10400 10873 11719 12403 12705 12950C 14110 14127 14766 14783 15043 15172 15301 15326 15884 16129 16183C 16189 16223 16249 16311 16519
G129	KT819230	Eastern Andalusia (Granada)	M1a3b	73 195 263 309insCC 315insC 489 750 813 1438 2706 4769 6446 6671 6680 7028 8701 8860 9039 9540 10398 10400 10475 10873 11719 12403 12414 12705 12950C 13637 14110 14766 14783 15043 15301 15326 15930 16129 16182C 16183C 16189 16249 16311 16519
G128	KT819231	Eastern Andalusia (Granada)	M1b2c	73 195 263 309insC 315insC 489 750 1007 1438 2706 3736 4769 6446 6680 7028 7258 8472 8512 8701 8860 9540 10398 10400 10873 10895 11719 12403 12705 12950C 12968 13111 14110 14766 14783 15043 15301 15326 16129 16182C 16183C 16189 16223 16249 16311 16399 16519
G132	KT819232	Eastern Andalusia (Granada)	U6a6b1	73 263 315insC 750 1438 2706 3348 4769 5471 7028 7391 7805 8407A 8557C 8860 11084 11467 11719 12308 12372 12501 12618 13440 13984 14179 14766 15326 16172 16219 16278 16290
G131	KT819233	Eastern Andalusia (Granada)	U6a1b2	73 195 263 309insC 315insC 750 1438 2706 3348 4769 6018 7028 7805 8860 10364 11467 11719 12308 12372 14179 14562 14766 14927 15326 16172 16219 16235 16278 16294
G130	KT819234	Eastern Andalusia (Granada)	U6a3b	73 146 152 185 188 263 309insCC 315insC 750 1211 1438 2706 3348 4769 7028 7805 8860 11268 11467 11719 12308 12372 13431 14179 14766 15326 15634 15790 16172 16179 16183C 16189 16219 16278
G116	KT819235	Eastern Andalusia (Granada)	U6a7a1	73 152 263 315insC 523delAC 750 794A 1193 1438 1692T 2706 3348 4769 5120 5471 7028 7805 8167 8473 8860 11467 11719 12308 12372 14179 14766 15043 15326 15530 15632 16172 16219 16278
G134	KT819236	Eastern Andalusia (Granada)	U6b	73 259 263 315insC 508 523delAC 750 1438 2706 3348 4225 4769 7028 8860 9438 11467 11719 12308 12372 14766 15326 15601 16172 16219 16311 16519
A02	KT819237	Morocco Berber (Asni)	L1b1a6	73 152 182 185T 195 247 263 315insC 357 523delAC 709 710 750 769 825A 1018 1738 2352 2706 2758 2768 2885 3308 3594 3666 3693 4104 4164 4769 5036 5046 5393 5655 6548 6827 6989 7028 7055 7146 7256 7389 7521 7867 8248 8468 8655 8701 8835 8860 9540 9755 10398 10688 10810 10873 11719 12519 12705 13105 13506 13650 13789 13880A 14110 14178 14203 14560 14766 14769 15115 15326 16187 16189 16223 16264 16270 16278 16293 16311 16519
A108	KT819238	Morocco Berber (Asni)	L2a1	73 143 146 152 195 263 315insC 750 769 1018 1438 1628 2416 2706 2789 3594 4104 4769 5460 7028 7175 7256 7274 7521 7771 8206 8701 8860 9221 9540 10115 10398 10873 11719 11914 11944 12693 12705 13590 13623 13650 13803 14566 14766 15301 15326 15747 15784 16093 16189 16223 16278 16294 16309 16390 16519
A27	KT819239	Morocco Berber (Asni)	L2b1a	73 150 152 182 195 198 204 207 260 263 309insC 315insC 418 523delAC 750 769 1018 1438 1442 1706 2332 2358 2416 2706 3594 4104 4158 4370 4767 4769 5027 5331A 5814 6026 6713 7028 7256 7521 7624A 8080 8206 8387 8701 8860 8939 9221 9540 9663 10115 10398 10828 10873 11654 11719 11944 12236 12705 12793 12948 13590 13650 13814 13924 14003 14059 14766 15110 15217 15301 15326 16093 16114A 16129 16148 16213 16223 16278 16355 16362 16368 16390
A107	KT819240	Morocco Berber (Asni)	L2b1a2	73 150 152 182 195 198 263 315insC 418 523delAC 590insA 750 769 1018 1118 1438 1442 1706 2332 2358 2416 2706 3594 4104 4158 4370 4767 4769 5027 5331A 5814 6026 6713 7028 7256 7521 7569 7624A 8080 8206 8387 8701 8860 9221 9540 10115 10398 10828 10873 11719 11944 12007 12236 12705 12948 13590 13650 13924 14059 14180 14766 15110 15217 15301 15326 16114A 16129 16213 16223 16278 16355 16362 16390

A18	KT819241	Morocco Berber (Asni)	L3e5	73 150 263 309insC 315insC 398 523delAC 1438 2352 2706 4769 7028 8392 8701 8860 9540 10398 10819 10873 11719 12705 14212 14766 15301 15326 16041 16223 16355 16519
A78	KT819242	Morocco Berber (Asni)	L3e5a	73 150 152 242 263 309insC 315insC 398 523delAC 1438 2352 2706 4769 7028 8392 8701 8860 9540 10398 10751 10819 10873 11719 12705 13317 13934 14212 14766 14798 15301 15326 16041 16124 16223 16519
A07	KT819243	Morocco Berber (Asni)	U6a7a1	73 152 263 315insC 750 794A 1193 1438 1692T 2706 3348 4769 5120 5471 7028 7805 8473 8860 9389 11467 11719 12308 12372 14179 14766 15043 15326 15530 15632 16172 16219 16278
B67	KT819244	Morocco Berber (Bouhria)	L1b1a	73 152 182 185T 195 247 263 309insC 315insC 357 523delAC 709 710 750 769 825A 1018 1738 2352 2706 2758 2768 2885 3308 3594 3666 3693 4104 4769 5036 5046 5393 5655 6548 6827 6989 7028 7055 7146 7256 7389 7521 7867 8248 8468 8655 8701 8860 9540 10398 10688 10810 10873 11719 12519 12705 13105 13506 13650 13789 13880A 14178 14203 14560 14766 14769 15115 15326 16093 16126 16187 16189 16223 16264 16270 16278 16311 16519
B86	KT819245	Morocco Berber (Bouhria)	L1b1a	73 152 182 185T 195 247 263 309insC 315insC 357 523delAC 709 710 750 769 825A 1018 1738 2352 2706 2758 2768 2885 3308 3594 3666 3693 4104 4769 5036 5046 5393 5655 6548 6827 6989 7028 7055 7146 7256 7389 7521 7867 8248 8468 8655 8701 8860 9540 10398 10688 10810 10873 11719 12519 12705 13105 13506 13650 13789 13880A 14178 14203 14560 14766 14769 15115 15326 16093 16126 16187 16189 16223 16264 16270 16278 16311 16519
B126	KT819246	Morocco Berber (Bouhria)	L1b1a6	73 152 182 185T 195 247 263 309insC 315insC 357 523delAC 709 710 750 769 825A 1018 1738 2352 2706 2758 2768 2885 3308 3594 3666 3693 4104 4769 5036 5046 5393 5655 6548 6827 6989 7028 7055 7146 7256 7389 7521 7867 8248 8468 8655 8701 8860 9540 9755 10398 10688 10810 10873 11719 12519 12705 13105 13506 13650 13789 13880A 14110 14178 14203 14560 14766 14769 15115 15262 15326 16126 16187 16189 16223 16264 16270 16278 16293 16311 16519
B120	KT819247	Morocco Berber (Bouhria)	L2a1k	73 143 146 152 195 263 315insC 750 769 1018 1438 2416 2706 2789 3594 4104 4769 6722 7028 7175 7256 7274 7521 7771 8206 8701 8860 9221 9540 10115 10398 10873 11719 11914 11944 12693 12705 13590 13650 13803 14566 14766 15064 15301 15326 15784 16129 16189 16192 16223 16278 16294 16309 16390 16519
B28	KT819248	Morocco Berber (Bouhria)	L2a1c	73 143 146 152 195 263 309insC 315insC 523delAC 750 769 1018 1438 2416 2706 2789 3010 3594 4104 4769 6164 6663 7028 7175 7256 7274 7521 7679 7771 8065 8206 8701 8860 9221 9320 9540 10115 10398 10873 11719 11914 11944 12693 12705 13488 13590 13650 13803 14002 14566 14766 15301 15326 15784 16223 16278 16294 16390 16519
B130	KT819249	Morocco Berber (Bouhria)	L3e5a	73 150 263 315insC 398 523delAC 1438 2352 2706 4769 7028 8392 8701 8860 9540 10398 10819 10873 11719 12705 13317 14212 14766 15301 15326 16041 16223 16519
B21	KT819250	Morocco Berber (Bouhria)	U6a8a	73 143 263 309insC 315insC 1438 2706 3348 4769 7028 7805 8062 8282 8860 10172 11467 11539 11719 12308 12372 14179 14766 15326 16172 16183C 16189 16219 16278
Fig05	KT819251	Morocco Berber (Figuig)	L1b1a8	73 146 152 182 185T 195 247 263 315insC 357 523delAC 709 710 750 769 825A 1018 1738 2352 2706 2758 2768 2885 3308 3594 3666 3693 4104 4769 5036 5046 5393 5655 6548 6827 6989 7028 7055 7146 7256 7298 7389 7521 7867 8248 8468 8655 8701 8860 9540 10398 10688 10810 10873 11719 12519 12705 13105 13506 13650 13789 13880A 14178 14203 14560 14766 14769 15115 15326 16126 16187 16189 16223 16264 16278 16293 16311 16519
Fig39	KT819252	Morocco Berber (Figuig)	L1b1a6	73 152 182 185T 195 198 247 263 309insC 315insC 357 523delAC 709 710 750 769 825A 1018 1738 2352 2706 2758 2768 2885 3308 3594 3666 3693 4104 4769 5036 5046 5393 5655 6548 6827 6989 7028 7055 7146 7256 7389 7521 7867 8248 8468 8655 8701 8860 9540 9755 10398 10688 10810 10873 11719 12519 12705 13105 13506 13650 13789 13880A 14110 14178

				14203 14305 14560 14766 14769 15115 15326 15903 16093 16126 16172 16187 16189 16223 16264 16270 16278 16293 16311 16519
Fig08	KT819253	Morocco Berber (Figuig)	L2a1k	73 143 146 152 195 263 315insC 750 769 1018 1438 2416 2706 2789 3594 4104 4769 6722 7028 7175 7256 7274 7521 7771 8206 8701 8860 9221 9540 10115 10398 10873 11719 11914 11944 12693 12705 13590 13650 13803 14566 14766 15301 15326 15784 16041 16129 16189 16192 16223 16278 16294 16309 16390
Fig69	KT819254	Morocco Berber (Figuig)	L2a1k	73 143 146 152 195 263 315insC 750 769 1018 1438 2416 2706 2789 3594 4104 4769 6722 7028 7175 7256 7274 7521 7771 8206 8701 8860 9221 9540 10115 10398 10873 11719 11914 11944 12693 12705 13590 13650 13803 14566 14766 15301 15326 15784 16041 16129 16189 16192 16223 16278 16294 16309 16390
Fig84	KT819255	Morocco Berber (Figuig)	L2e	73 146 152 182 199 263 309insCC 315insC 479 719 750 769 1018 1211 1438 2416 2706 3537 3594 4104 4205 4481 4512 4562 4769 5069T 5228 6014 6261 7028 7256 7521 8206 8383 8522 8701 8860 9221 9377 9380 9540 9635 9971 10115 10398 10873 11719 11930 11935 12189 12441 12705 13590 13650 13708 14299 14766 15301 15326 15697 15734 15889 15930 16111A 16145 16184 16189 16223 16239 16259 16278 16355 16390 16399 16400 16519
Fig13	KT819256	Morocco Berber (Figuig)	L3b1	73 151 152 263 315insC 523delAC 750 1438 2071 2706 3450 4769 5417 5773 6221 7028 8701 8860 9174 9449 9540 10086 10373 10398 10601 10873 11380 11719 12705 13105 13914A 14766 15301 15311 15314 15326 15824 15944delT 16124 16223 16234 16278 16362 16409 16519
Fig14	KT819257	Morocco Berber (Figuig)	L3b1a9	73 152 263 315insC 523delAC 750 1438 2706 3450 4769 5773 6221 7028 8701 8860 9449 9540 10086 10373 10398 10873 11002 11719 12705 13105 13914A 14766 15301 15311 15326 15824 15944delT 16051 16223 16256 16278 16362 16519
Fig115	KT819258	Morocco Berber (Figuig)	L3b1a3	73 263 309insC 315insC 503 523delAC 750 1193 1438 2706 3450 4769 5773 6221 7028 8251 8701 8860 9449 9540 10086 10373 10398 10873 11002 11719 12705 13105 13914A 13933 14766 15301 15311 15326 15824 15944delT 16124 16223 16278 16311 16362 16519
Fig06	KT819259	Morocco Berber (Figuig)	L3b1a5	73 152 263 309insC 315insC 480 499 523delAC 750 1438 1780 2706 3450 4769 5063 5773 6221 7028 8701 8860 9449 9540 9615 10086 10373 10398 10700 10873 11002 11719 12612 12705 13105 13914A 14766 15301 15311 15326 15824 15944delT 16124 16223 16278 16293 16362 16519
Fig03	KT819260	Morocco Berber (Figuig)	L3e2b	73 150 152 195 263 315insC 750 1438 1676 2352 2706 3834 4769 6956 7028 8701 8848 8860 9540 10398 10819 10873 11719 12705 13263 14212 14766 14905 15301 15326 16172 16182C 16183C 16189 16223 16320 16519
Fig09	KT819261	Morocco Berber (Figuig)	L3e2b1a2	73 150 152 195 263 315insC 750 1438 2352 2483 2706 3277 4769 4811 5899delC 7028 8701 8860 9377 9540 10398 10819 10873 11719 12406 12432 12705 14212 14766 14905 15301 15326 16172 16183C 16189 16223 16320 16519
Fig04	KT819262	Morocco Berber (Figuig)	L3e5a	73 150 152 263 309insC 315insC 398 523delAC 1438 2352 2706 4769 7028 8392 8701 8860 9102 9540 10398 10819 10873 11719 12705 13317 13934 14212 14766 15301 15326 16041 16223 16519
Fig18	KT819263	Morocco Berber (Figuig)	L3e5a1	73 150 152 263 315insC 398 523delAC 1438 2352 2706 2833 4769 7028 8392 8701 8860 9540 10398 10819 10873 11719 12705 13317 14073 14212 14766 15301 15326 16041 16188 16223 16519
Fig72	KT819264	Morocco Berber (Figuig)	M1a1b2	73 195 207 263 315insC 489 750 813 930 1438 1820 2706 3705 4769 6446 6671 6680 7028 7853 8537 8701 8860 9540 10398 10400 10873 11719 12346 12403 12705 12950C 14110 14766 14783 14788 15043 15301 15326 15497 16129 16189 16223 16249 16311 16359 16519

Fig92	KT819265	Morocco Berber (Figuig)	U6a7a1	73 152 263 315insC 750 794A 1193 1438 1692T 2706 3348 4769 5120 5471 7028 7805 8473 8860 11467 11719 12308 12372 14179 14766 15043 15326 15530 15632 16172 16219 16278
Fig01	KT819266	Morocco Berber (Figuig)	U6a7c1	73 263 309insC 315insC 750 1438 2706 3348 4769 7028 7337 7805 8860 11467 11719 12308 12372 14766 15043 15326 16172 16182C 16183C 16189 16219 16278

Table B. TMRCA estimates of most common sub-clades of U6, M1 and L lineages

Haplogroup	ρ whole-mtDNA age estimate	ρ synonymous age estimate	Maximum Likelihood age estimate
**U6**	**35352 [24682 - 46435]***	**34529 [20161 - 48897]**	**35454 [23982 - 47404]**
U6a	25944 [20350 - 31670]	32121 [21535 - 42707]	25392 [20213 - 30685]
U6b	12472 [7416 - 17670]	7884 [3430 - 12337]	11328 [7724 - 15005]
U6d	13206 [7035 - 19586]	14376 [3431 - 25322]	12034 [6940 - 17272]
U6c	10820 [5286 - 16531]	9096 [0 - 18380]	9924 [5013 - 14976]
**M1**	**26280 [17939 - 34916]**	**26902 [12069 - 41735]**	**26137 [18651 - 33861]**
M1a	21224 [15123 - 27497]	19493 [10209 - 28777]	20996 [16503 - 25582]
M1b	20259 [12644 - 28150]	17139 [8225 - 26052]	22416 [14810 - 30287]
**L1**	**122789 [98301 - 147841]**	**124149 [89583 - 158715]**	**138413 [103499 - 174245]**
L1b	29278 [15961 - 43323]	37084 [11807 - 62360]	35757 [17744 - 54974]
L1c	91163 [73724 - 109057]	93524 [70066 - 116982]	87211 [70058 - 104831]
**L2**	**77931 [57873 - 98728]**	**85997 [51183 - 120810]**	**96869 [77126 - 117149]**
L2a	58685 [37826 - 80637]	80119 [39501 - 120738]	79306 [59869 - 99423]
L2b	29383 [19627 - 39522]	29839 [14528 - 45150]	26271 [20284 - 32409]
L2c	17261 [13993 - 20582]	15768 [11911 - 19624]	20291 [16429 - 24223]
L2d	18261 [10105 - 26748]	23237 [5831 - 40642]	14347 [7984 - 20926]
L2e	35805 [26454 - 45467]	45333 [28298 - 62367]	35929 [26074 - 46131]
**L3**	**54012 [45043 - 63190]**	**58241 [44289 - 72192]**	**68179 [56232 - 80425]**
L3a	48295 [34860 - 62255]	57816 [34780 - 80851]	56442 [42270 - 71127]
L3b	19373 [12383 - 26599]	28289 [9423 - 47156]	23719 [14005 - 33858]
L3d	30640 [22589 - 38944]	36553 [24576 - 48529]	33296 [24372 - 42518]
L3c	9245 [2343 - 16433]	15768 [315 - 31220]	10108 [2128 - 18467]
L3f	48466 [33507 - 64079]	43234 [22990 - 63478]	53297 [40936 - 66065]
L3e	33669 [25738 - 41832]	35551 [22994 - 48108]	39970 [29738 - 50550]
L3i	33538 [21464 - 46161]	46091 [23412 - 68769]	45326 [30929 - 60361]
L3k	27266 [13741 - 41573]	30222 [8520 - 51923]	27260 [15724 - 39359]
L3x	36534 [26272 - 47170]	33037 [18769 - 47306]	38823 [28227 - 49801]
L3h	72146 [54007 - 90944]	76496 [48708 - 104284]	64029 [52049 - 76330]

**Age estimates in years with 95% confidence intervals. Negative lower values were ignored*

Table C. TaqMan design for genotyping U6 and L/M lineages

Nucleotide position	Haplogroup			Sequence (5'-3')	Final concentration (µM)
3348	U6	primers	3311F	CCATGGCCAACCTCCTACTC	0.9
			3381R	TCGTTCGGTAAGCATTAGGAATG	0.9
		probes	allele A	***VIC***-ATTGTACCCATTCTAATC	0.2
			allele G	***FAM***-ATTGTACCCATTCTGAT	0.2
10873	L or M	primers	10837F	CACAACCACCCACAGCCTAATT	0.9
			10981R	GGGTAGGAGTCAGGTAGTTAGTATTAGGA	0.9
		probes	allele T	***VIC***-CATCCCTCTACTATT	0.2
			allele C	***FAM***-TCATCCCCCTACTATTTT	0.2

Table D. HVS-I compilation for mtDNA comparative analyses. Geographic affiliations are as follows: *IB* (Iberia), *EUR* (Europe), *CME* (Central Mediterranean Europe), *EEU* (Eastern Europe), *ANA* (Anatolia), *NES* (Near East), *ARA* (Arabian Peninsula), *SWAs* (Southwestern Asia), *NAF* (North Africa), *WAF* (West Africa), *CAF* (Central Africa), *SWA* (Southwestern Africa), *EAF* (East Africa), *SEA* (Southeastern Africa), *SAF* (South Africa), *MAD* (Madagascar). *N is the sample size.

Code	Region	Population	Reference	N*
1	*IB*	Galicia	Richards et al. 2000; González et al. 2003	136
2	*IB*	León	Larruga et al. 2001	61
3	*IB*	Cantabria	Maca-Meyer et al. 2003	88
4	*IB*	Cantabria Pasiegos	Maca-Meyer et al. 2003; Cardoso et al. 2010	143
5	*IB*	Basque country	Richards et al. 2000	163
6	*IB*	Zamora	Álvarez et al. 2010	214
7	*IB*	*Andalusia (Huelva)*	*present study*	280
8	*IB*	Andalusia (Córdoba)	Casas et al. 2006	108
9	*IB*	*Andalusia (Granada)*	*present study*	470
10	*IB*	Canary Islands	Rando et al. 1999	300
11	*IB*	Ibiza	Picornell et al. 2005	50
12	*IB*	Majorca	Picornell et al. 2005	93
13	*IB*	Portugal (North)	González et al. 2003; Pereira et al. 2004	271
14	*IB*	Portugal (Center)	González et al. 2003; Pereira et al. 2004	317
15	*IB*	Portugal (South)	González et al. 2003; Pereira et al. 2004	260
16	*IB*	Azores	Brehm et al. 2003; Santos et al. 2003	235
17	*IB*	Madeira	Brehm et al. 2003	88
18	*EUR*	France	Dubut et al. 2004	210
19	*EUR*	Corsica (South)	Falchi et al. 2006	53
20	*EUR*	Austria (West)	Richards et al. 2000	101
21	*EUR*	Slovenia	Malyarchuk et al. 2003	103
22	*EUR*	Slovakia	Malyarchuk et al. 2008	207
23	*EUR*	Hungary	Irwin et al. 2007	416
24	*CME*	Italy (Torino)	Turchi et al. 2008	50
25	*CME*	Italy (Tuscany)	Richards et al. 2000; Falchi et al. 2006; Achilli et al. 2007; Turchi et al. 2008	480
26	*CME*	Italy (Ancona)	Turchi et al. 2008	73
27	*CME*	Italy (Roma)	Babalini et al. 2005; Turchi et al. 2008	110
28	*CME*	Italy (South)	Babalini et al. 2005	103
29	*CME*	Italy (Basilicata)	Ottoni et al. 2009a	92
30	*CME*	Italy (Calabria)	Ottoni et al. 2009a	95
31	*CME*	Sardinia (North)	Falchi et al. 2006	50
32	*CME*	Sardinia (Center)	Falchi et al. 2006	52
33	*CME*	Sardinia	Richards et al. 2000	69
34	*CME*	Sardinia (South)	Falchi et al. 2006	133
35	*CME*	Sicily	Ottoni et al. 2009a	154
36	*EEU*	Bosnia	Malyarchuk et al. 2003	144
37	*EEU*	Greece (North)	Irwin et al. 2008	319

38	*EEU*	Cyprus	Irwin et al. 2008	91
39	*ANA*	Turkey	Richards et al. 2000	271
40	*NES*	Lebanon	Badro et al. 2013	980
41	*NES*	Syria	Badro et al. 2013	234
42	*NES*	Druze Galilee	Shlush et al. 2008	185
43	*NES*	Jordan	González et al. 2009	101
44	*NES*	Palestine	Badro et al. 2013	120
45	*NES*	Iraq	Al-Zahery et al. 2003	52
46	*ARA*	Saudi Arabia (West)	Abu-Amero et al. 2008	59
47	*ARA*	Saudi Arabia (South)	Abu-Amero et al. 2008	88
48	*ARA*	Yemen	Kivisild et al. 2004; Non et al. 2011	210
49	*ARA*	Yemen (Tihama)	Cerný et al. 2008	58
50	*ARA*	Yemen (Ta'izz)	Cerný et al. 2008	68
51	*ARA*	Saudi Arabia (Center)	Abu-Amero et al. 2008	220
52	*ARA*	Dubai	Alshamali et al. 2008	249
53	*SWAs*	Iran	Metspalu et al. 2004	436
54	*NAF*	Saharawi	Plaza et al. 2003	56
55	*NAF*	Morocco (Souss)	Brakez et al. 2001	50
56	*NAF*	Morocco Berber (Asni)	Coudray et al. 2009	53
57	*NAF*	Morocco (Marrakech)	Falchi et al. 2006	52
58	*NAF*	Morocco (El Jadida)	Harich et al. 2010	81
59	*NAF*	Morocco	Rando et al. 1998; Turchi et al. 2009	145
60	*NAF*	Morocco (Tetouan)	Rhouda et al. 2009	209
61	*NAF*	Morocco (Al Hoceima)	Rhouda et al. 2009	79
62	*NAF*	Morocco Berber (Bouhria)	Coudray et al. 2009	70
63	*NAF*	Morocco (Oujda)	Rhouda et al. 2009	189
64	*NAF*	Morocco Berber (Figuig)	Coudray et al. 2009	94
65	*NAF*	Algeria	Bekada et al. 2013	240
66	*NAF*	Tunisia (Testour)	Cherni et al. 2009	50
67	*NAF*	Tunisia	Cherni et al. 2009; Turchi et al. 2009	115
68	*NAF*	Tunisia (Zriba)	Cherni et al. 2005	50
69	*NAF*	Tunisia (Jerba Island)	Loueslati et al. 2006	59
70	*NAF*	Libya	Fadhlaoui-Zid et al. 2011	269
71	*NAF*	Libyan Tuaregs	Ottoni et al. 2009b	129
72	*NAF*	Egypt Berber (Siwa)	Coudray et al. 2009	78
73	*NAF*	Egypt (Alexandria)	Saunier et al. 2009	277
74	*NAF*	Egypt	Krings et al. 1999	66
75	*NAF*	Egypt (South)	Krings et al. 1999	80
76	*WAF*	Cabo Verde	Brehm et al. 2002	292
77	*NAF*	Mauritania	González et al. 2006	64
78	*WAF*	Senegal	Watson et al. 1997; Rando et al. 1998; Stefflova et al. 2009	280
79	*WAF*	Sierra Leone	Jackson et al. 2005	276
80	*WAF*	Mali	Ely et al. 2006; González et al. 2006	204
81	*WAF*	Ghana	Veeramah et al. 2010	238
82	*CAF*	Nigeria (Center)	Watson et al. 1997	112
83	*CAF*	Nigeria (South)	Montano et al. 2013	129

84	*CAF*	Nigeria (Southeast)	Veeramah et al. 2010	1192
85	*CAF*	Nigeria (Northeast)	Cerný et al. 2007	69
86	*CAF*	Chad	Cerný et al. 2007	127
87	*CAF*	Cameroon (North)	Cerný et al. 2007	244
88	*CAF*	Cameroon (Northwest)	Veeramah et al. 2010	256
89	*CAF*	Cameroon (West)	Quintana-Murci et al. 2008	199
90	*CAF*	São Tomé	Mateu et al. 1997; Trovoada et al. 2003	153
91	*CAF*	Gabon (West)	Quintana-Murci et al. 2008	158
92	*CAF*	Gabon (Northwest)	Quintana-Murci et al. 2008	50
93	*CAF*	Gabon (North)	Quintana-Murci et al. 2008	66
94	*CAF*	Gabon (Center)	Quintana-Murci et al. 2008	146
95	*CAF*	Gabon (East)	Quintana-Murci et al. 2008	154
96	*CAF*	Gabon (Southeast)	Quintana-Murci et al. 2008	247
97	*CAF*	Gabon (Southwest)	Quintana-Murci et al. 2008	52
98	*CAF*	Congo	Montano et al. 2013	101
99	*SWA*	Angola (Cabinda)	Beleza et al. 2005	110
100	*SWA*	Angola (Southwest)	Coelho et al. 2009	365
101	*EAF*	Sudan	Soares et al. 2012	102
102	*EAF*	Sudan (South)	Krings et al. 1999	76
103	*EAF*	Ethiopia	Kivisild et al. 2004; Soares et al. 2012	347
104	*EAF*	Ethiopia (East)	Boattini et al. 2013	167
105	*EAF*	Ethiopia (Nyangatom)	Poloni et al. 2009	112
106	*EAF*	Kenya (Turkana)	Boattini et al. 2013	51
107	*EAF*	Kenya (North)	Boattini et al. 2013	69
108	*EAF*	Kenya (Maasai)	Boattini et al. 2013	81
109	*EAF*	Kenya	Watson et al. 1997; Brandstätter et al. 2004	329
110	*EAF*	Somalia	Soares et al. 2012	148
111	*EAF*	Tanzania (North)	Tishkoff et al. 2007	278
112	*EAF*	Tanzania (Center)	Knight et al. 2003	100
113	*SEA*	Zambia	de Filippo et al. 2010	78
114	*SEA*	Zimbawe	Castrì et al. 2009	59
115	*SEA*	Mozambique	Pereira et al. 2001; Salas et al. 2002	416
116	*SAF*	South Africa (Schmidtsdrift)	Chen et al. 2000	74
117	*SAF*	South Africa	Quintana-Murci et al. 2010	445
118	*MAD*	Madagascar	Tofanelli et al. 2009	133

References

Abu-Amero KK et al. (2008) *BMC Evol Biol* 8:45
Achilli A et al. (2007) *Am J Hum Genet* 80:759–768.
Alshamali F et al. (2008) *Forensic Sci Int Genet* 2:e9–e10
Álvarez L et al. (2010) *Am J Phys Anthr* 142:531–539.
Al-Zahery N et al. (2003) *Mol Phylogenet Evol* 28:458–472.
Babalini C et al. (2005) *Eur J Hum Genet* 13:902–912.
Badro DA et al. (2013) *PLoS One* 8:e54616.
Bekada A et al. (2013) *PLoS One* 8:e56775.
Beleza S et al. (2005) *Hum Genet* 117:366–375.
Boattini A et al. (2013) *Am J Phys Anthr* 150:375–385.
Brakez Z et al. (2001) *Ann Hum Biol* 28:295–307.
Brandstätter A et al. (2004) *Int J Leg Med* 118:294–306.
Brehm A et al. (2002) *Ann Hum Genet* 66:49–60.
Brehm A et al. (2003) *Hum Genet* 114:77–86.

Cardoso S et al. (2010) *J Forensic Sci* 55:1196–1201.
Casas MJ et al. (2006) *Am J Phys Anthr* 131:539–551.
Castrì L et al. (2009) *Am J Phys Anthr* 140:302–311.
Cerný V et al. (2007) *Ann Hum Genet* 71:433–452.
Cerný V et al. (2008) *Am J Phys Anthr* 136:128–137.
Chen YS et al. (2000) *Am J Hum Genet* 66:1362–1383.
Cherni L et al. (2005) *Hum Biol* 77:61–70.
Cherni L et al. (2009) *Am J Phys Anthr* 139:253–260.
Coelho M et al. (2009) *BMC Evol Biol* 9:80.
Coudray C et al. (2009) *Ann Hum Genet* 73:196–214.
Dubut V et al. (2004) *Eur J Hum Genet* 12:293–300.
Ely B et al. (2006) *BMC Biol* 4:34.
Fadhlaoui-Zid K et al. (2011) *Am J Phys Anthr* 145:107–117.
Falchi A et al. (2006) *J Hum Genet* 51:9–14.
De Filippo C et al. (2010) *Am J Phys Anthr* 141:382–394.
González AM et al. (2003) *Am J Phys Anthr* 120:391–404.
González AM et al. (2006) *Ann Hum Genet* 70:631–657.
González AM et al. (2009) *Ann Hum Biol* 35:212–231.
Harich N et al. (2010) *BMC Evol Biol* 10:138.
Irwin J et al. (2007) *Int J Leg Med* 121:377–383.
Irwin J et al. (2008) *Int J Leg Med* 122:87–89.
Jackson BA et al. (2005) *Am J Phys Anthr* 128:156–163.
Kivisild T et al. (2004) *Am J Hum Genet* 75:752–770.
Knight A et al. (2003) *Curr Biol* 13:464–473.
Krings M et al. (1999) *Am J Hum Genet* 64:1166–1176.
Larruga JM et al. (2001) *Eur J Hum Genet* 9:708–716.
Loueslati BY et al. (2006) *Am J Hum Biol* 18:149–153.
Maca-Meyer N et al. (2003) *Ann Hum Genet* 67:329–339.
Malyarchuk BA et al. (2003) *Ann Hum Genet* 67:412–425.
Malyarchuk BA et al. (2008) *Ann Hum Genet* 72:228–240.
Mateu E et al. (1997) *Ann Hum Genet* 61:507–518.
Metspalu M et al. (2004) *BMC Genet* 5:26.
Montano V et al. (2013) *BMC Evol Biol* 13:24.
Non AL et al. (2011) *Am J Phys Anthr* 144:1–10.
Ottoni C et al. (2009a) *Ann Hum Biol* 36:785–811.
Ottoni C et al. (2009b) *Ann Hum Genet* 73:438–448.
Pereira L et al. (2001) *Ann Hum Genet* 65:439–458.
Pereira L et al. (2004) *Int J Leg Med* 118:132–136.
Picornell A et al. (2005) *Am J Phys Anthr* 128:119–130.
Plaza S et al. (2003) *Ann Hum Genet* 67:312–328.
Poloni ES et al. (2009) *Ann Hum Genet* 73:582–600.
Quintana-Murci L et al. (2008) *Proc Natl Acad Sci U S A* 105:1596–1601.
Quintana-Murci L et al. (2010) *Am J Hum Genet* 86:611–620.
Rando JC et al. (1998) *Ann Hum Genet* 62:531–550.
Rando JC et al. (1999) *Ann Hum Genet* 63:413–428.
Rhouda T et al. (2009) *Mitochondrion* 9:402–407.
Richards M et al. (2000) *Am J Hum Genet* 67:1251–1276.
Salas A et al. (2002) *Am J Hum Genet* 71:1082–1111.
Santos C et al. (2003) *Ann Hum Genet* 67:433–456.
Saunier JL et al. (2009) *Forensic Sci Int Genet* 3:e97–e103.
Shlush LI et al. (2008) *PLoS One* 3:e2105.
Soares P et al. (2012) *Mol Biol Evol* 29:915–927.
Stefflova K et al. (2009) *PLoS One* 4:e7842.
Tishkoff SA et al. (2007) *Mol Biol Evol* 24:2180–2195.
Tofanelli S et al. (2009) *Mol Biol Evol* 26:2109–2124.
Trovoada MJ et al. (2003) *Ann Hum Genet* 68:40–54.
Turchi C et al. (2008) *Int J Leg Med* 122:199–204.
Turchi C et al. (2009) *Forensic Sci Int Genet* 3:166–172.
Veeramah KR et al. (2010) *BMC Evol Biol* 10:92.
Watson E et al. (1997) *Am J Hum Genet* 61:691–704.

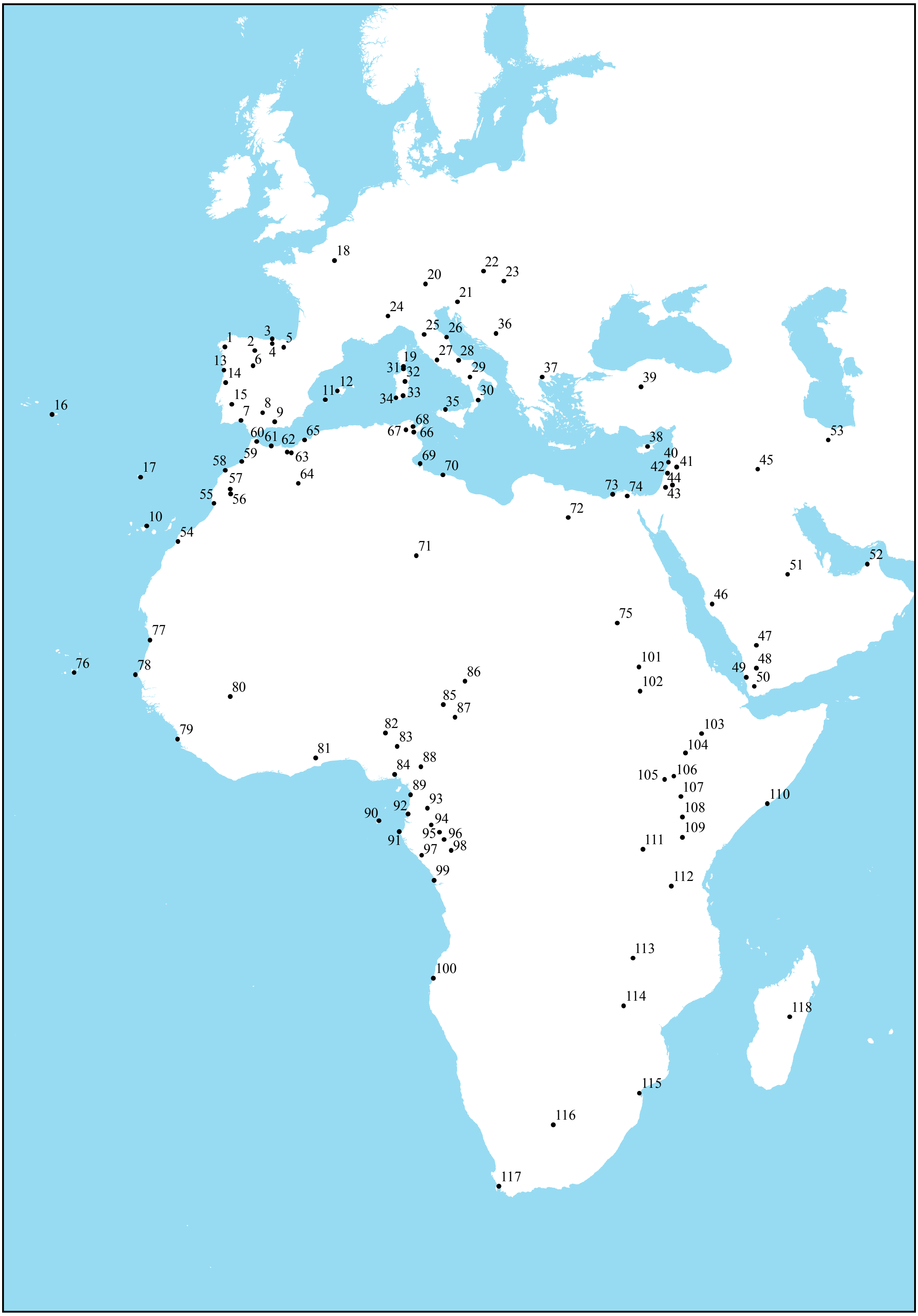

Figure A. Geographic location of the populations used for haplogroup frequency surface maps. Codes and references in Table D.

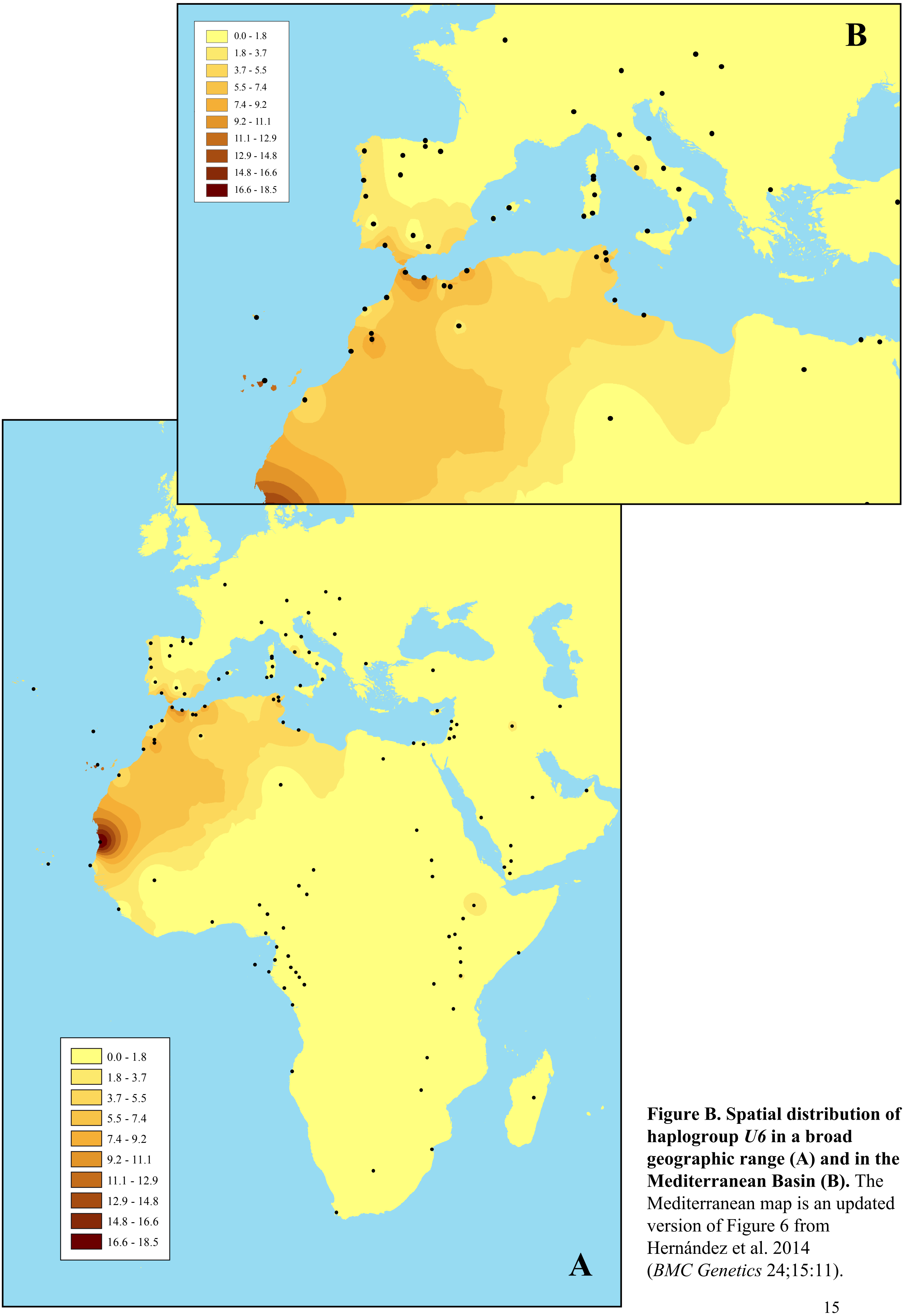

Figure B. Spatial distribution of haplogroup *U6* in a broad geographic range (A) and in the Mediterranean Basin (B). The Mediterranean map is an updated version of Figure 6 from Hernández et al. 2014 (*BMC Genetics* 24;15:11).

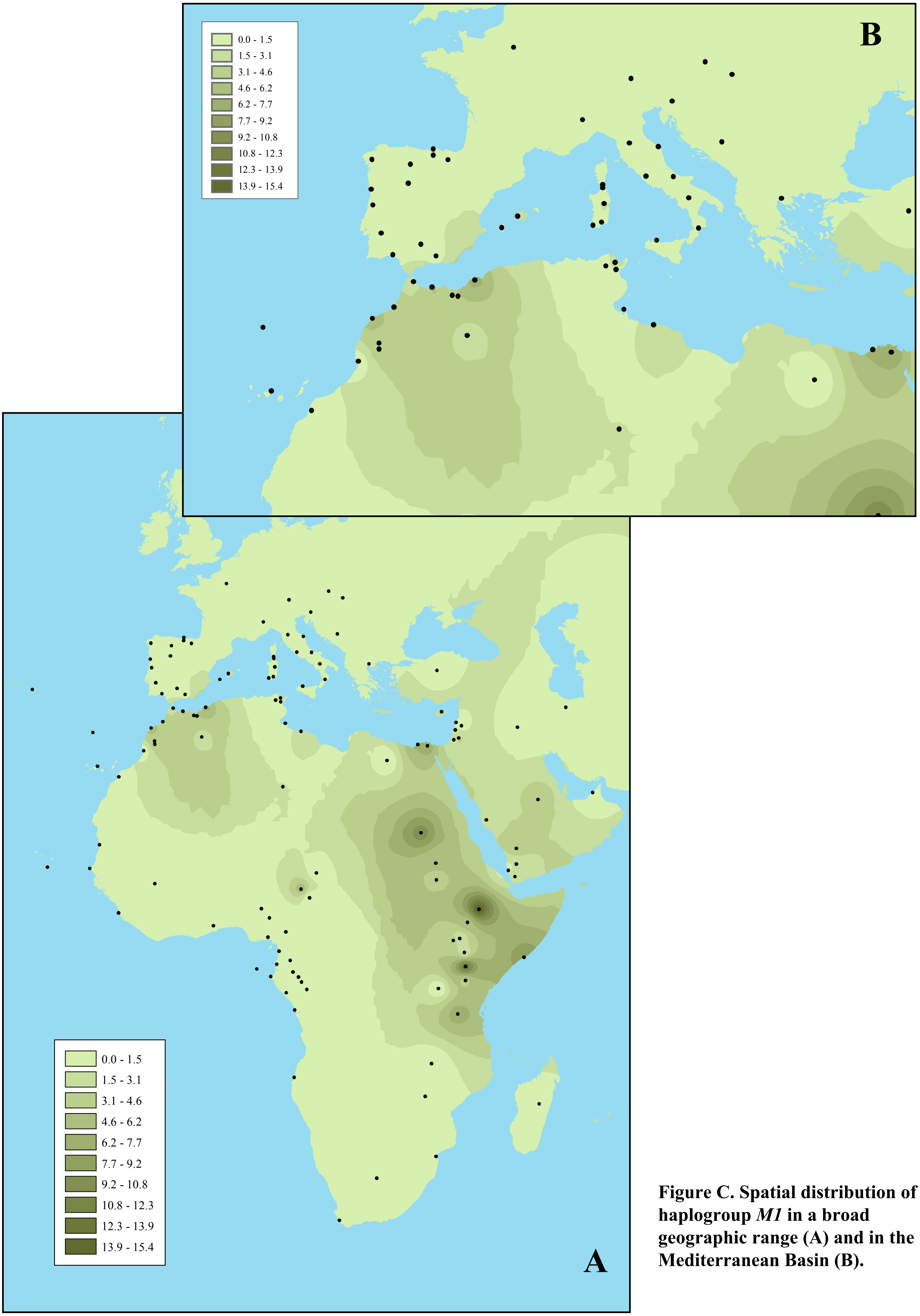

Figure C. Spatial distribution of haplogroup *M1* in a broad geographic range (A) and in the Mediterranean Basin (B).

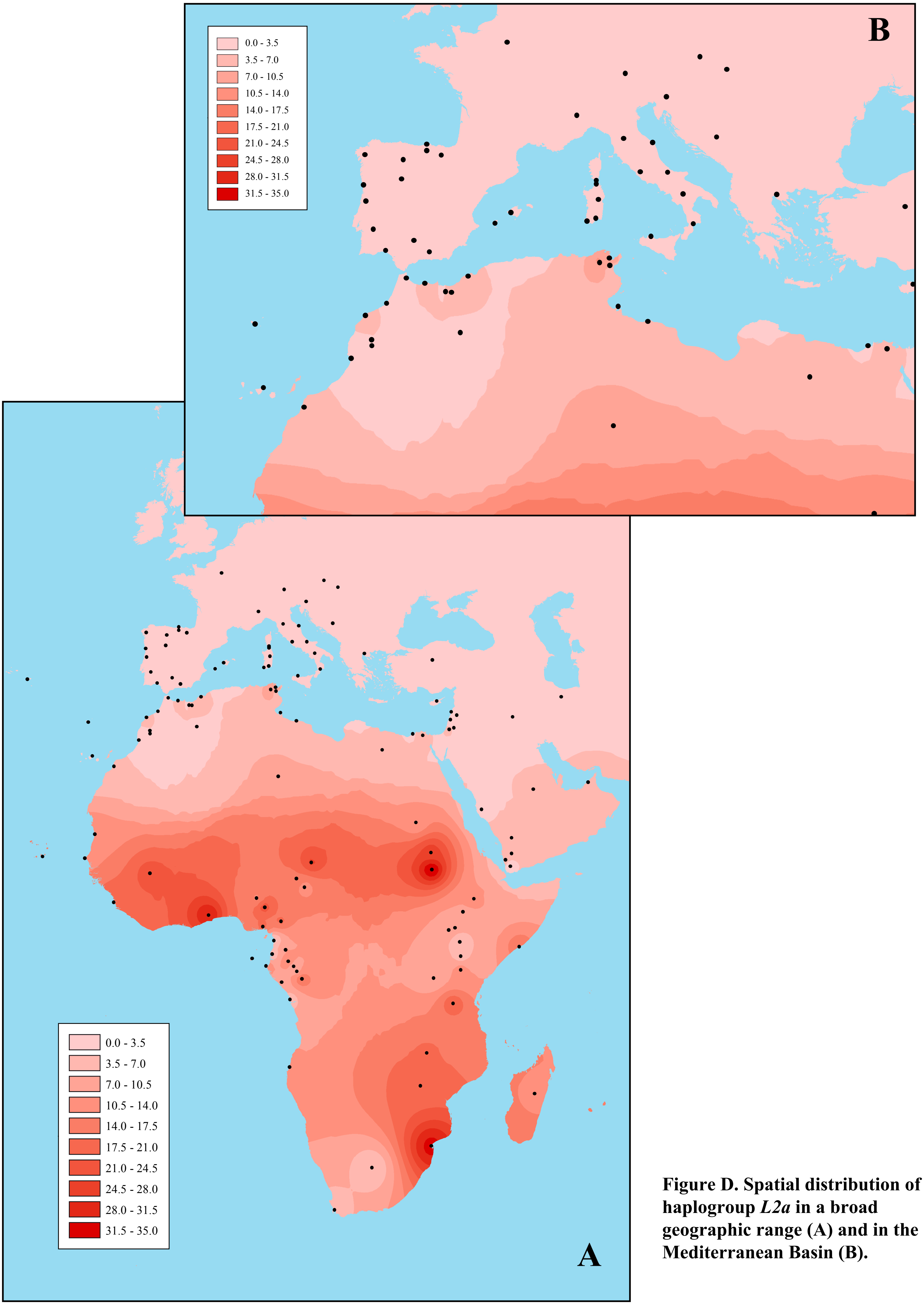

Figure D. Spatial distribution of haplogroup *L2a* in a broad geographic range (A) and in the Mediterranean Basin (B).

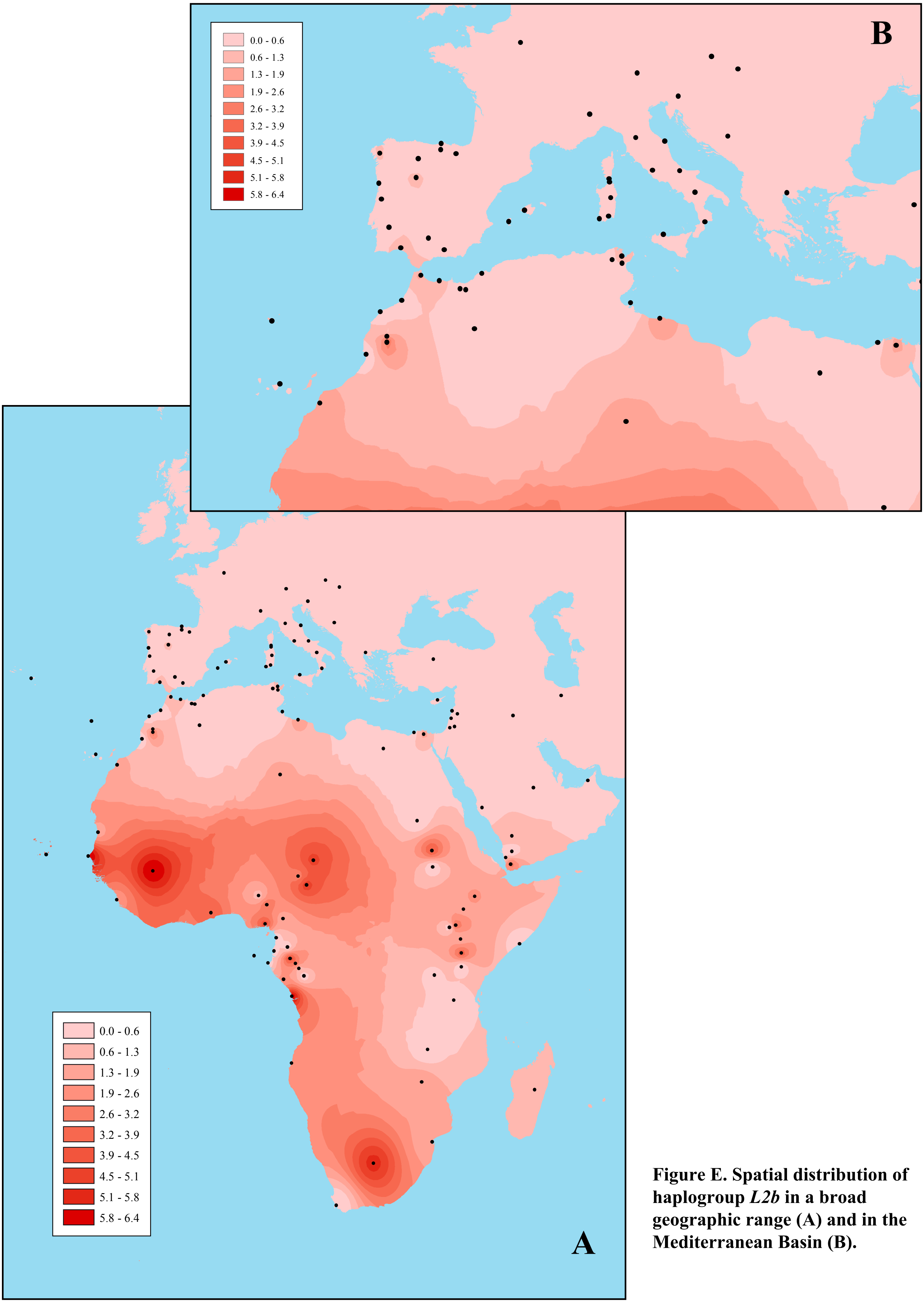

Figure E. Spatial distribution of haplogroup *L2b* in a broad geographic range (A) and in the Mediterranean Basin (B).

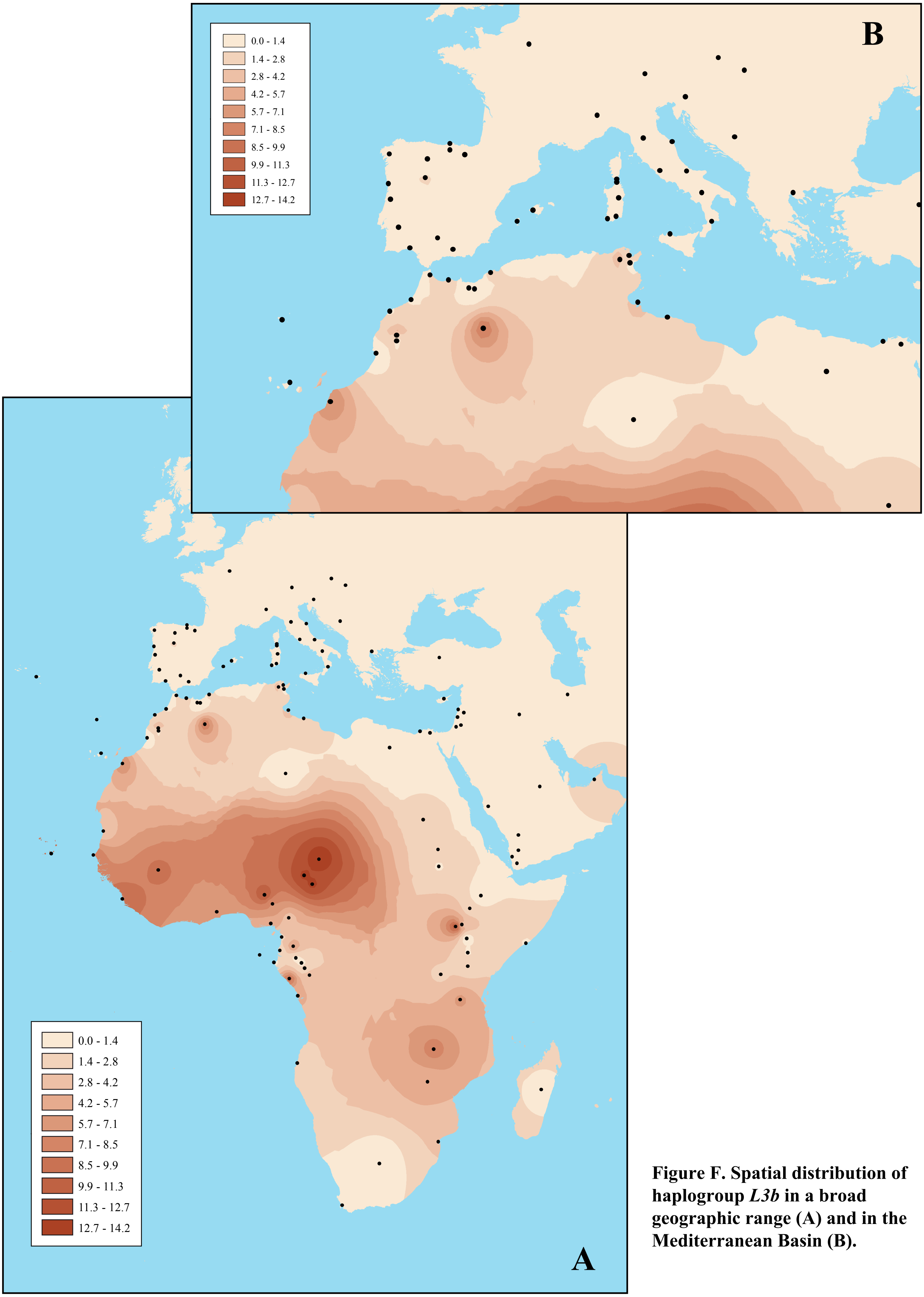

Figure F. Spatial distribution of haplogroup *L3b* in a broad geographic range (A) and in the Mediterranean Basin (B).

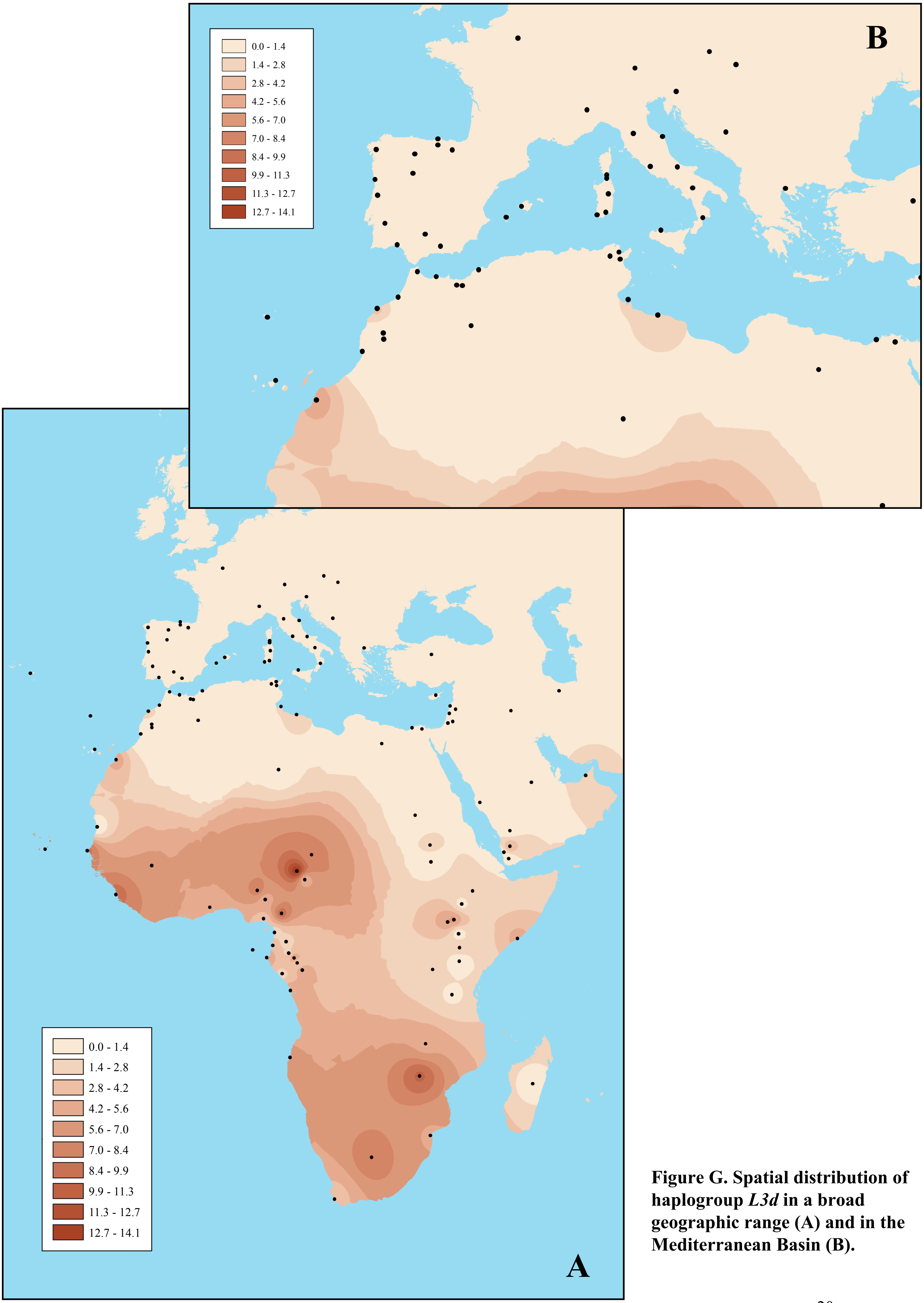

Figure G. Spatial distribution of haplogroup *L3d* in a broad geographic range (A) and in the Mediterranean Basin (B).

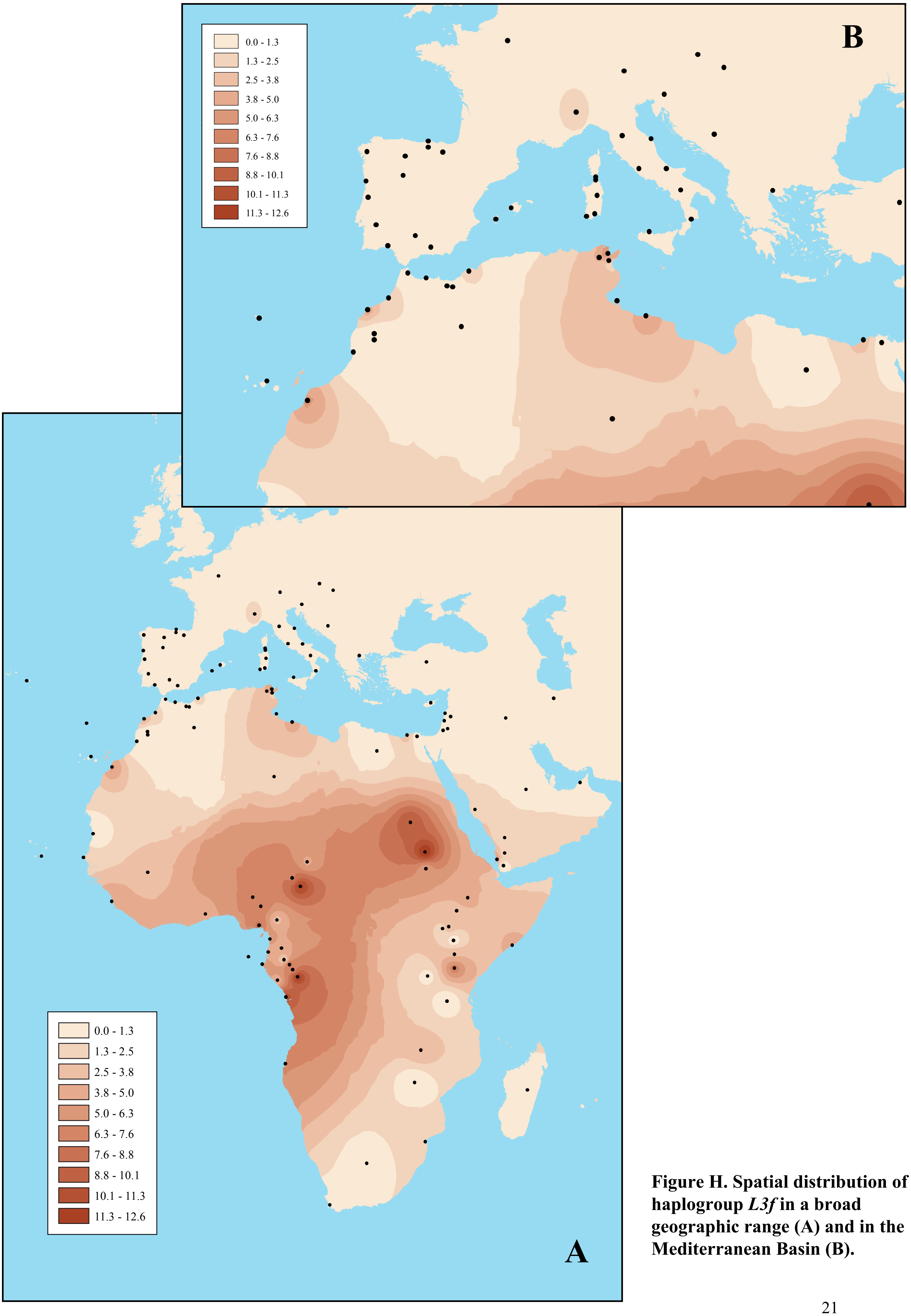

Figure H. Spatial distribution of haplogroup *L3f* in a broad geographic range (A) and in the Mediterranean Basin (B).

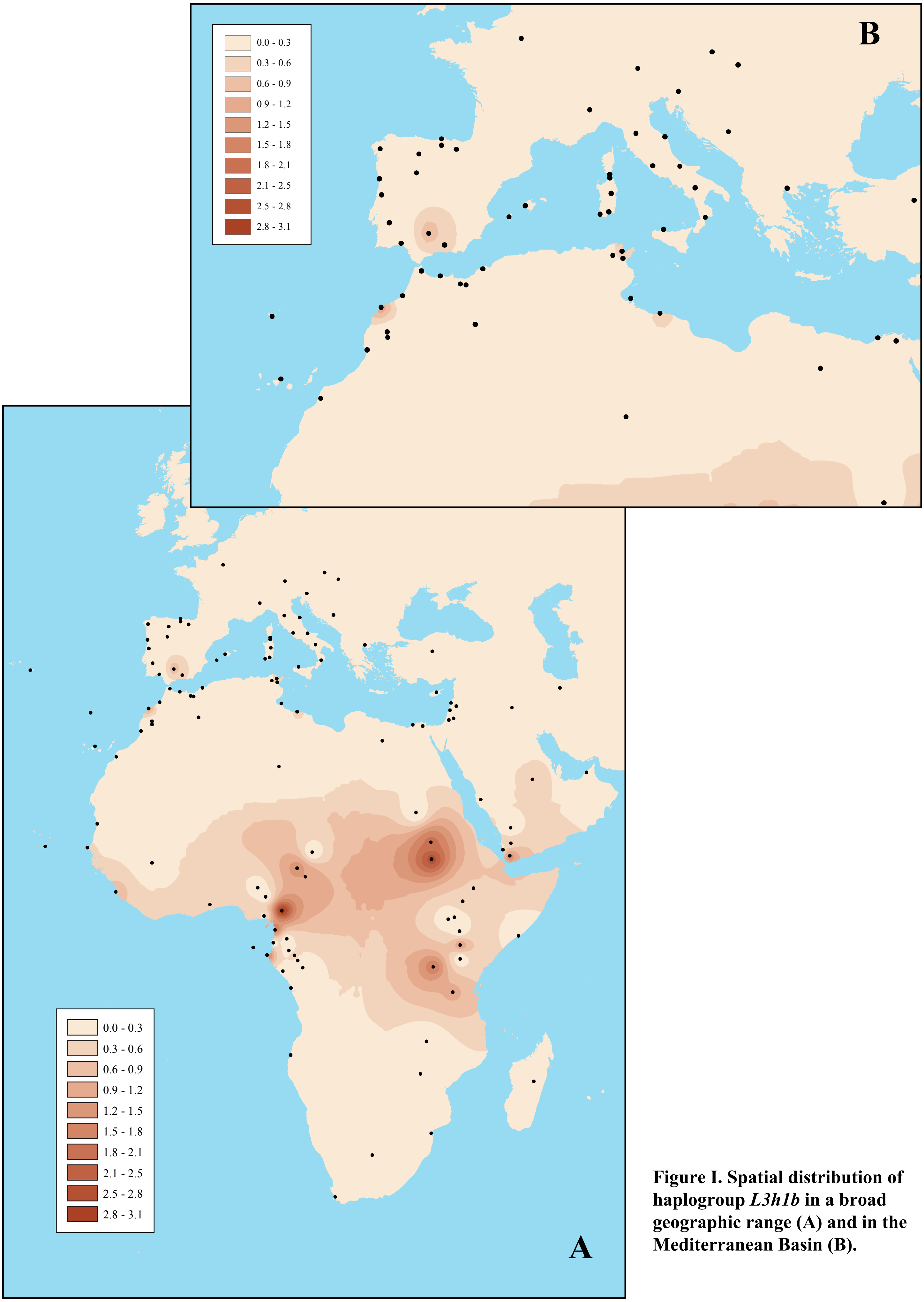

Figure I. Spatial distribution of haplogroup *L3h1b* in a broad geographic range (A) and in the Mediterranean Basin (B).

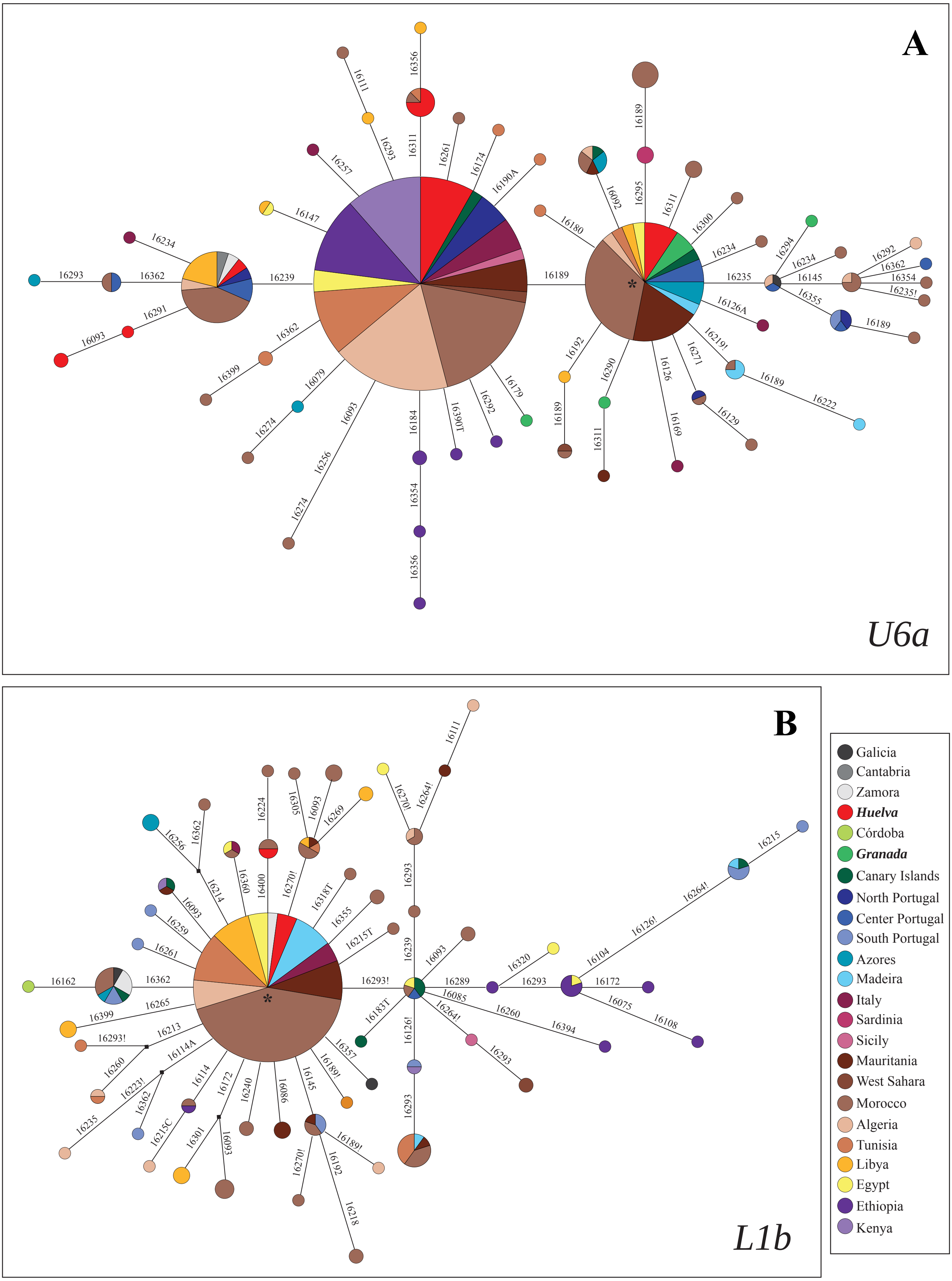

Figure J. Specific median-joining networks for lineages *U6a* and *L1b* found in Iberia, Italy and Africa. M-J genealogies are based on 209 (*U6a*) and 175 (*L1b*) HVS-I sequences. Asterisks indicate basal nodes with the following mutations respect to the rCRS: 16172-16219-16278 (*U6a*) and 16126-16187-16189-16223-16264-16270-16278-16293-16311 (*L1b*). See population details and references in Table D.

Figure K. Probabilistic proportion of founder clusters considering two migration events (at 0.5 and 8.0 ka), using *f1* and *f2* criteria, for migrations of L lineages into North Africa, Iberia and Mediterranean Europe.

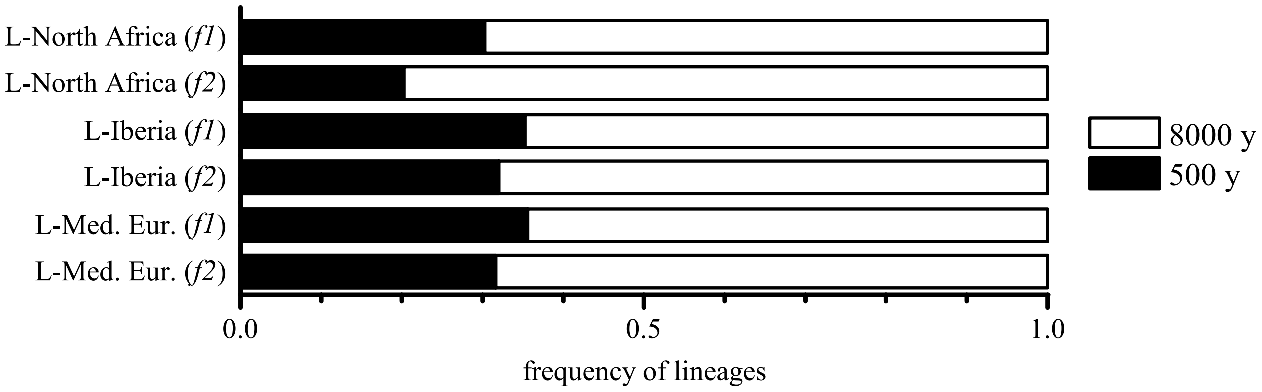

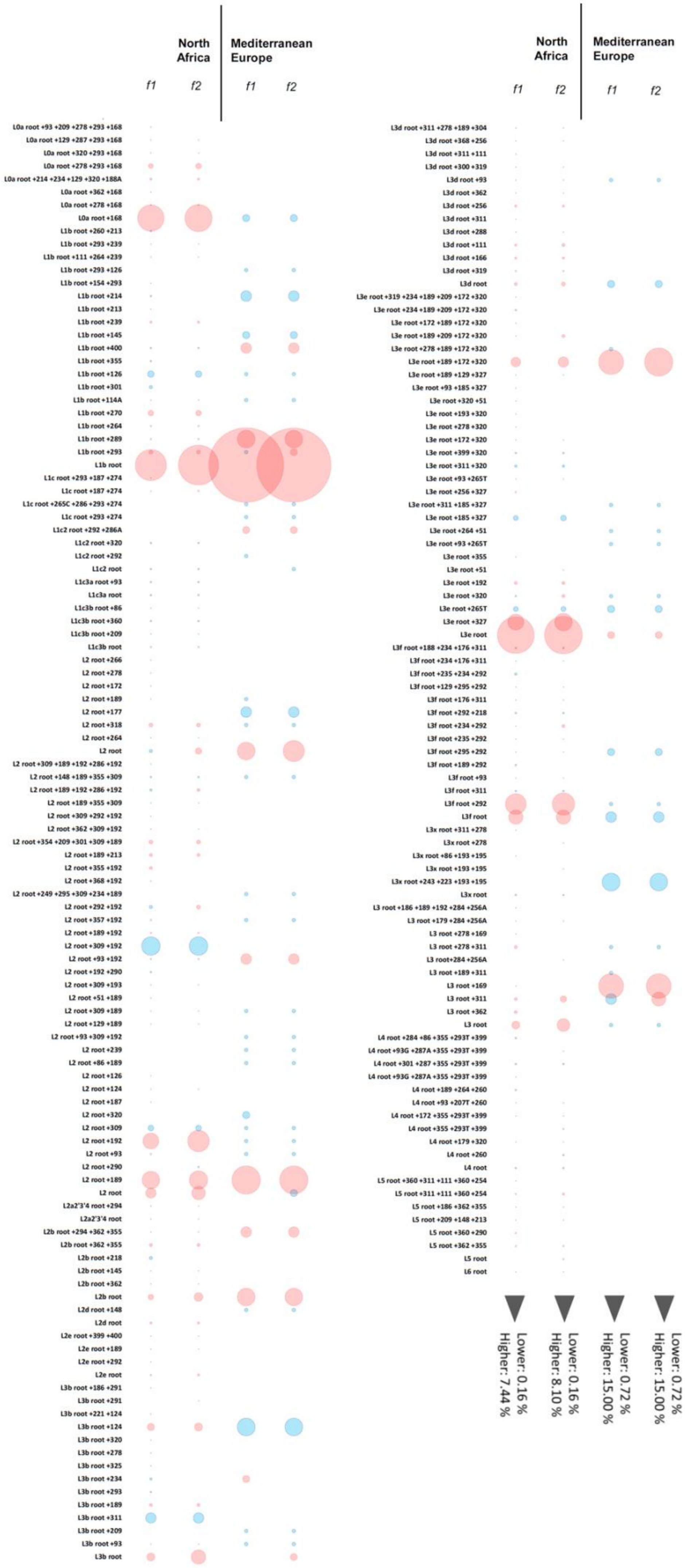

Figure L. Haplogroup L founders introduced into North Africa and Mediterranean. Red circles indicate clades probably introduced during early Holocene and blue circles indicate clades probably introduced in historic times in each region. The size of each circle is proportional to the frequency of each founder within the total L haplogroup founders in the analyses for the two regions with two founder selection criteria (*f1** and *f2*). The highest and lowest frequencies for each analysis are indicated in the Figure.

* Founders must have at least one (*f1*) or two (*f2*) derived branches in the source population.
